# Accessing real-time interaction between antimicrobial liposomes and live *Staphylococcus epidermidis* using surface plasmon resonance microscopy

**DOI:** 10.1016/j.biosx.2026.100792

**Published:** 2026-05-16

**Authors:** Kam Hang Chan, Nancy Diaz Batista, Kevin Diego-Perez, Adaly Garcia, Rachelle Soriano, Maria Kala, Juan Luis Cazares, Sheehan Belleca, Stellina Ao, Christina Dhoj, Zhipeng Dai, Sam On Ho, Gary Fujii, Edith Porter, Yixian Wang

**Affiliations:** a Department of Chemistry and Biochemistry, California State University, Los Angeles, Los Angeles, CA, 90032, USA; b Department of Biological Sciences, California State University, Los Angeles, Los Angeles, CA, 90032, USA; c Molecular Express, Inc., Rancho Dominguez, CA, 90220, USA

**Keywords:** Antimicrobial lipids, Cholesteryl linoleate, Liposomes, *Staphylococcus epidermidis*, Surface plasmon resonance microscopy, Scanning electron microscopy, Atomic force microscopy

## Abstract

*Staphylococcus epidermidis* (SE) is a Gram-positive bacterium that is a major cause of healthcare-associated infections (HAIs), largely due to its ability to form biofilms on medical devices and its resistance to many antibiotics. Cholesteryl linoleate (CL) is a lipid secreted by epithelial cells that has been shown to have antimicrobial effects against SE, but how it interacts with the bacteria remains poorly understood. In this study, we developed a surface plasmon resonance microscopy (SPRm) protocol to observe the interaction between CL formulated in phospholipid (CL-PL) liposomes and live stationary phase SE cells in real time. Buffer control experiments showed that the bacteria remained viable throughout the SPRm procedure. Our results provide evidence that CL directly binds to surface of SE and showed that CL-PL binding to SE is concentration-dependent and much more pronounced than binding of PL only. The binding was not uniform but instead varied from one SE cell region to another, with some cell regions showing strong binding and others showing much less indicative of a heterogenous bacterial population as expected for stationary phase bacteria. This work supports further exploration of CL and similar lipids to treat antibiotic-resistant infections caused by SE and other pathogens associated with HAIs, and presents a novel approach of studying antimicrobial activities capable to discern heterogeneity within live bacterial populations.

## 1. Introduction

Healthcare-associated infections (HAIs) have become a growing concern, with *Staphylococcus epidermidis* (SE) identified as a major contributor to catheter-related bloodstream infections, accounting for approximately 30% of such cases (Chabi and Momtaz, 2019; Eiff et al., 1998). SE is a common Gram-positive bacterium populating the skin and adjacent mucosal surfaces as an important member of the normal microbiota. The high prevalence among HAIs is attributed to SE–s ability to form robust biofilms on plastic medical devices, such as catheters (Peng et al., 2022; Pouget et al., 2023). When these devices are inserted into the circulation, SE can expand along the devices directly into the bloodstream. Additionally, SE involved in HAIs often exhibits significant antibiotic resistance, making conventional treatments less effective (Chabi and Momtaz, 2019; Eladli et al., 2019; Ventola, 2015; Xu et al., 2020). Thus, novel approaches to combat SE infections are needed. Building on natural host defense mechanisms may lead to the development of effective preventive or therapeutic treatments. One example is to exploit epithelial cell mediated host defense.

We have previously found that lipids, including cholesteryl esters, secreted by epithelial cells are antimicrobial (Do et al., 2008) and that the cholesteryl ester cholesteryl linoleate (CL) inhibits growth of SE (Cheung Lam et al., 2016). Dissecting how CL interacts with live SE would provide valuable insights into designing alternative CL based antimicrobial strategies. Surface plasmon resonance microscopy (SPRm) provides a robust platform for studying such molecular interactions with live cells. SPRm combines SPR, a powerful and sensitive optical technique used to study molecular interactions in real-time without requiring labels or dyes, with microscopic visualization (Bocková et al., 2019; Zhou et al., 2020). SPR has been employed to study the kinetics of cholesterol interactions with cholesterol-binding proteins (He et al., 2022) and the interactions between antimicrobial agents and bacteria, such as the binding of pleuromutilin derivatives to *Staphylococcus aureus*, another Gram-positive bacterium that shares the same genus with SE (Zhang et al., 2021). However, these studies used a traditional SPR approach that averages signals across the entire sample, thereby lacking spatial resolution for visualizing interaction with bacteria populations. SPRm integrates SPR with microscopy and enables the visualization of specific binding sites, the detection of sample heterogeneity, facilitates efficient background noise subtraction from non-covered areas, and provides detailed insights into the interaction dynamics. Recent research demonstrated the use of SPRm to monitor bacterial adhesion, characterize binding interactions, and differentiate bacterial strains based on surface properties. These approaches provide valuable insights into host microbe interaction and may be useful in interrogating and infection mechanisms (Syal et al., 2015, 2016, 2017).

This study aimed to explore the interaction between CL and live SE using SPRm. We optimized the protocol of immobilizing live SE on SPRm sensing chips , demonstrated viability of immobilized SE throughout the SPRm procedure, and monitored real-time interaction between the antimicrobial lipid CL and live SE at spatially defined places. The relevance of this work extends to novel drug discovery as well as epithelial cell biology, as the measured interaction of CL with SE may have implications for the natural epithelial cell defenses. Understanding how CL interacts with SE could provide valuable insights into novel therapeutic strategies.

## 2. Experimental section

### 2.1. Chemicals and materials

1-Dodecanthiol, absolute ethanol, and 3-aminopropyltriethoxysilane (APTES), phosphate-buffered saline (PBS) tablets were purchased from Fisher Scientific (Waltham, MA) and used without further purification. Bovine serum albumin (BSA, fatty acid, nuclease and protease free) was purchased from Sigma-Aldrich (St. Louis, MO). Tryptic soy was purchased from Fisher Scientific and prepared as broth (TSB) or agar (TSA) according to the manufacturer’s instructions. All aqueous solutions were prepared using double-deionized water (dH_2_O, resistivity = 18.2 MΩ cm at 25 °C, Milli-Q Ultrapure water EQ 7000 Purification System, MilliporeSigma, Burlington, MA). Silicon wells were cut from flexiPERM reuseable silicon inserts (Sarstedt, Germany). Gold SPRm sensor chips were purchased from Biosensing Instrument (Tempe, AZ). These chips consist of an approximately 50 nm layer of Au deposited on top of a 2 nm Ti layer on a glass coverslip.

### 2.2. Liposome preparation and characterization

Liposomes were prepared via dry lipid film rehydration technique as previously described (Beadell et al., 2021) (See Section 1 in Supplementary Material for details). Liposomes were formulated to contain phospholipids only (HSPC:DSPG at 18:2 mg/mL; designated as PL) and phospholipids and cholesteryl linoleate (HSPC:DSPG:CL at 18:2:1 mg/mL, designated as CL-PL, concentration of CL at 1.54 mM). After filtration, the liposomes were characterized using dynamic light scattering (Zetasizer Nano, Malvern Panalytical, Malvern, UK). The zeta potential was −32.1 ± 0.9 mV for PL and −32.9 ± 3.3 mV for CL-PL. The initial size was 89.9 ± 2.4 nm for PL and 98.2 ± 1.9 nm for CL-PL. The size slightly increased during storage at 4 °C over the next few days, reaching 110.1 ± 3.3 nm for PL and 108.4 ± 1.1 nm for CL-PL on Day 8, but then stabilized measuring 112.2 ± 4.3 nm for PL and 110.4 ± 3.8 nm for CL-PL on Day 14 (values are averages ± S.D. of two measurements). The encapsulation efficiency of CL was determined by peak area ratios of HPLC (Shimadzu HPLC (LC-2030C plus)) chromatograms at 205 nm and found to be 91%.

### 2.3. Bacterial culture, immobilization, and characterization

For each experiment, *Staphylococcus epidermidis* (ATCC 700566) was cultured from a cryobead stock (CryoSavers^™^ Brucella Broth with 10% Glycerol and Beads, Hardy Diagnostics, Santa Maria, CA) (See Section 2 in Supplementary Material for details). A SPRm gold sensor chip was washed three times each with dH_2_O and ethanol, followed by annealing with hydrogen flame. The cleaned chip was then placed into a glass incubator containing 10 mL ethanol and 12 μL 1-dodecanethiol (DDT) and incubated overnight. It was then rinsed and submerged to a chip holder containing 1% (v/v) APTES dissolved in ethanol for 2 min and then rinsed and dried. For bacteria drop casting, 2 μL of the bacteria suspension was spotted on the gold sensor chip, which was then placed in a biosafety hood to air dry.

**Fig. 1.**
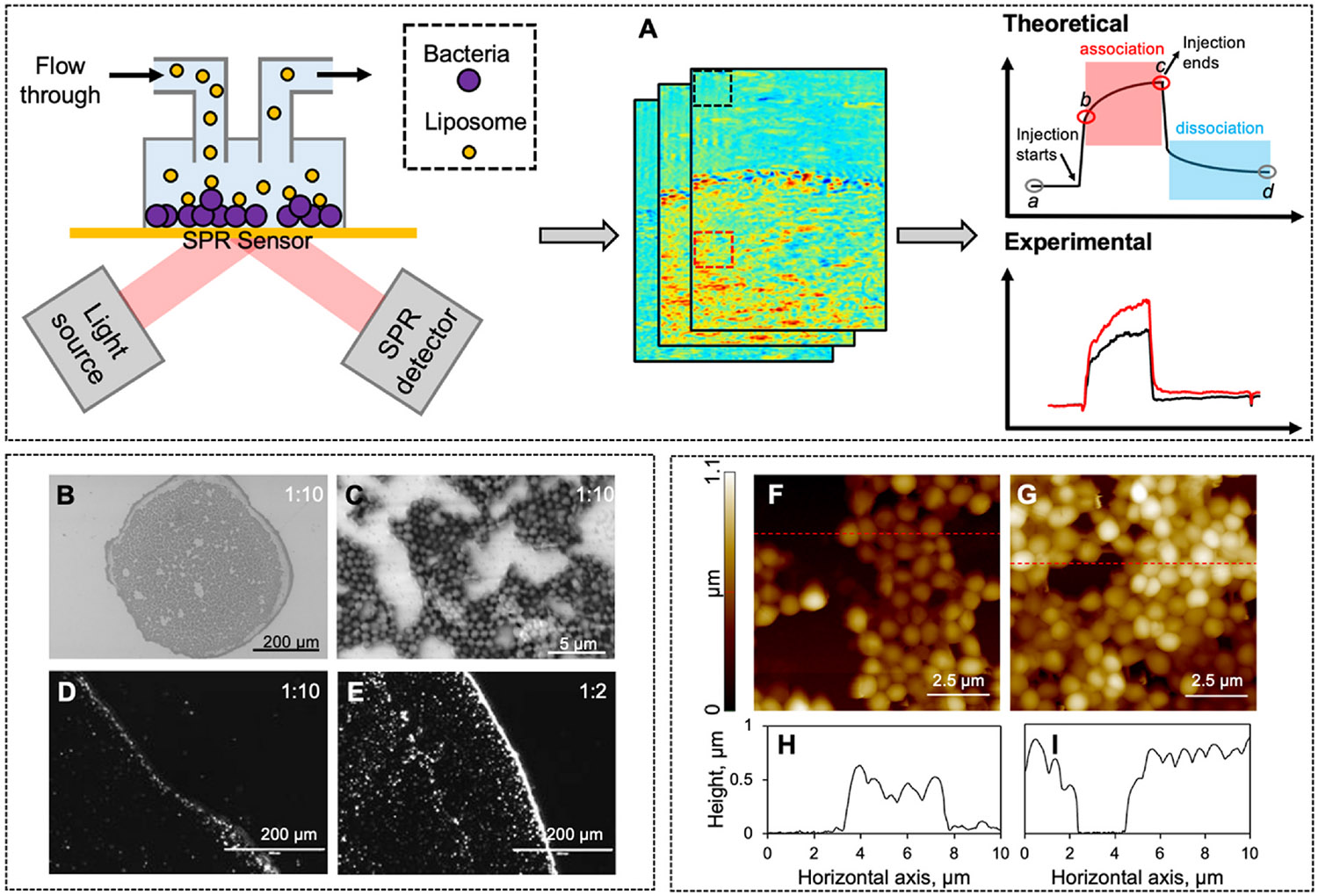
SPRm procedure and deposition of bacteria. (A) Schematic of the SPRm setup and the corresponding data visualization. (B-G) Microscopy of *Staphylococcus epidermidis* bacteria deposited onto chips. (B, C) Scanning electron microscopy with sensor chips (SEM) and (D, E) bright-field images with glass chips. (F, G) Atomic force microscopy (AFM) characterization of bacteria deposited on sensor chips with F showing a single layer region and G a multilayer region. (H, I) Corresponding height images collected from F (H) and G (I) at the locations indicated by the red dashed lines in F and G. For microscopy images B-D, 18-h cultures of bacteria were washed and diluted 1:10. For E-G, bacteria were diluted 1:2 prior to depositing.

The deposited samples were then characterized with either scanning electron microscopy (SEM, FEI Axia ChemiSEM LoVac, Thermo Scientific), optical microscopy (EVOS FL Color, Life Technologies, used in transmission mode) or atomic force microscopy (AFM, Park NX12, Park Systems, Seoul, South Korea) (Fig. 1). SEM was conducted under low vacuum mode with 20 Pa Pressure and 10 kV voltage. AFM was conducted using tapping mode with PPP-NCHR cantilevers (42 N/m, 330 kHz, Park Systems).

The viability of bacteria immobilized on glass cover slips or SPRm gold sensor chips was assessed using tetrazolium agar, immediately after spotting and after the SPRm procedure that was done with buffer (PBS supplemented with 0.25% BSA) only. Motility test medium with triphenyl tetrazolium chloride (Hardy Diagnostics, Santa Maria, CA) was reheated to liquify and poured into 6-well tissue culture plates (Corning, NY, about 3 mL per well) and resolidified. Samples were placed directly onto the tetrazolium agar surface with bacteria facing the agar and slightly pressed onto the agar to ensure contact. After a 24-h incubation at 37 °C, the agar media were inspected for color change in the region where bacteria had been deposited.

### 2.4. SPRm instrument setup

SPRm experiments were conducted using a surface plasmon resonance microscopy system (SPRm 200 Series, Biosensing Instruments, Tempe, AZ). As illustrated in Fig. 1, bacteria were immobilized on the gold sensor chip, which was mounted onto the prism stage with a refractive index-matching fluid. P-polarized light was directed onto the gold chip through the prism to induce plasmonic excitations, and variations in the reflected light intensity were captured by an SPR detector. A flow cell was loaded onto the sample to induce the flow of buffer containing the reagents. Specifically, 1 × PBS containing 0.25% BSA (PBS-BSA) was used as the running buffer. Three different concentrations of PL or CL-PL diluted from the original stocks (1 indicating a 1:1000, 2 a 1:200, and 3 a 1:40 dilution; for CL-PL corresponding to a CL concentration of 1.54, 7.70, and 38.5 μM, respectively) were injected in the order specified for each experiment. Each experiment was also accompanied by a blank injection using the running buffer and two calibration injections using 90% running buffer. During each injection, the sample was exposed to 400 μL of reagents at the flow rate of 200 μL/min. During each experiment, the SPRm image stacks were recorded.

### 2.5. Data processing

#### 2.5.1. Visualizing and quantifying the signal change across the sample surface

SPR image stacks were converted to .tif stacks using ImageAnalysis (Biosensing Instruments, Tempe, AZ) and then converted to image sequences using ImageJ 1.53c (Wayne Rasband, National Institutes of Health) for further analysis. MATLAB (R2019b; MathWorks) was then used to apply color coding to the images, enabling precise localization of potential binding events and visualization of binding intensity (code included in Supplementary Material, Section 7). Response data were extracted from individual regions of interest (ROIs) from SPRm measurements for quantifying the binding interactions (see Section 3 in Supplementary Material for data extraction details).

#### 2.5.2. Statistical analysis

The extracted numerical data was transferred to R Studio to create violin plots (code included in Supplementary Material, Section 8) that visualize the distribution and variability of binding activity within the bacterial populations and across CL concentrations. Statistical significance of observed differences was determined with IBM SPSS Statistics version 29.0.2.0 (20). To assess the difference in response units overall between bacteria-containing ROIs (bacteria) and ROIs free of bacteria (background), an Independent Samples T-test was employed. For all other comparisons with three or more groups, One-way ANOVA with Bonferroni post-hoc analysis was used.

#### 2.5.3. Kinetic analysis

ImageAnalysis was used to conduct kinetic fitting to the association (red) and dissociation (blue) regions within the sensorgrams (Fig. 1A) at every ROI using a 1:1 kinetic binding interaction model (Thirumurthy et al., 2026). Subsequently, rigorous filters were applied to eliminate outlier ROIs that produced invalid fitting results. The remaining valid ROIs are highlighted in red on the corresponding SPRm images to clearly map the cell-specific interactions. Simultaneously, isoaffinity scatter plots were generated, and histograms extracted from these plots were fitted with Gaussian distributions. Because the kinetic parameters span several orders of magnitude, the statistical analysis was performed on the base-10 logarithm of the values, and the final results are shown as the mean and 95% confidence interval (CI), obtained by taking the antilog. This approach ensured the accurate determination of the association rate (k_a_), dissociation rate (k_d_), and equilibrium dissociation constant (K_D_).

## 3. Results and discussions

### 3.1. Immobilization, characterization, and viability

We first optimized the bacteria immobilization process. The dual-layer modification with DDT and APTES enabled stable bacterial attachment, minimizing signal fluctuations during SPRm experiments (Liu et al., 2020). The low-magnification SEM image (Fig. 1B) shows a circular region covered with immobilized bacteria (about 2 × 10^5^ bacteria in 2 μL of a 1:10 dilution deposited) with a sharp and well-defined boundary. A zoomed-in SEM image (Fig. 1C) revealed individual spherical bacteria packing on the surface as an approximately 70% confluent monolayer. We then investigated the reproducibility of the coverage density with the more readily available bright field microscope (Fig. 1D and E). However, the bacteria deposited from a 1:10 diluted suspension (Fig. 1D) were sparsely distributed and the bacteria containing surface area was barely distinguishable from the adjacent background surface area except at the outer edge of the dried drop. In contrast, bacteria from a 1:2 diluted suspension (Fig. 1E, about 1 × 10^6^ bacteria in 2 μL deposited) produced a density similar to what had been observed with SEM in the previous experiment. Thus, there was a day-to-day variation in sample preparation, and we decided to proceed with a 1:2 dilution of the washed bacteria for deposition in all subsequent experiments to ensure we always have sufficient bacterial density.

The cellular height is a critical parameter for SPRm analysis, as the decay length of the surface plasmon resonance field is approximately 200 nm, with a practical detection limit (95% field decay) of around 500 nm (Peterson et al., 2014). Since SE is a spherical bacterium, its height roughly corresponds to its diameter which appeared to be approximately 600 nm according to the SEM image shown in Fig. 1C. However, AFM provides more accurate determination of the height (Fig. 1F-I). An analysis of over ten individual cells revealed an average height of 639 ± 62 nm. It should be noted that the bacterial samples were dried for both the SEM and AFM imaging; therefore, the fully hydrated size of the bacteria in an aqueous medium would reach the μm scale. Based on the AFM measurements, for a single monolayer of bacteria (Fig. 1F and H), roughly one-fifth to one-third of the cell volume resides within the highly sensitive evanescent field region. Previous studies have demonstrated that SPRm effectively captures detectable signals from binding events occurring at these cellular regions within the evanescent field (Wang et al., 2012). The upper portion of the bacteria continues to contribute to the overall signal, albeit at an exponentially reduced level. Conversely, in regions containing multiple bacterial layers (Fig. 1G and I), the aggregate height vastly exceeds the evanescent field–s penetration depth. Because the binding sites within the sensitive evanescent region are physically blocked by the stacked cells, these multilayered areas are expected to exhibit significantly lower signal changes.

Another critical aspect for developing a real time interaction analysis between antimicrobial compounds and live bacteria is that the bacteria’s viability is not negatively impacted by the SPRm procedure itself. Traditional fluorescence-based dyes such as BCECF-AM and Calcein-AM, and the redox based colorigenic/fluorogenic resazurin (Trinh and Lee, 2022) were tested but these failed to indicate the viability of SE in our experimental setting (Fig. S1). Alternatively, tetrazolium, a redox-based dye, incorporated in agar medium such as motility agar provides a simple and direct way to visualize metabolic activity of live bacteria (Ishiyama, 1997). The suitability of this medium for our experiment was first verified by inoculating SE into the agar (Fig. S2) and further evaluated with chip-deposited SE immediately after deposition and after a full run of SPRm imaging with buffer only (Fig. S3). While this assay does not allow making any conclusions regarding bacterial functionality, it confirmed that the bacteria remained metabolically active and viable throughout the SPRm run with buffer only, and we next assessed the real time interaction of CL-PL and PL by SPRm.

### 3.2. SPRm monitoring of the interaction between liposomes and live SE

**Fig. 2.**
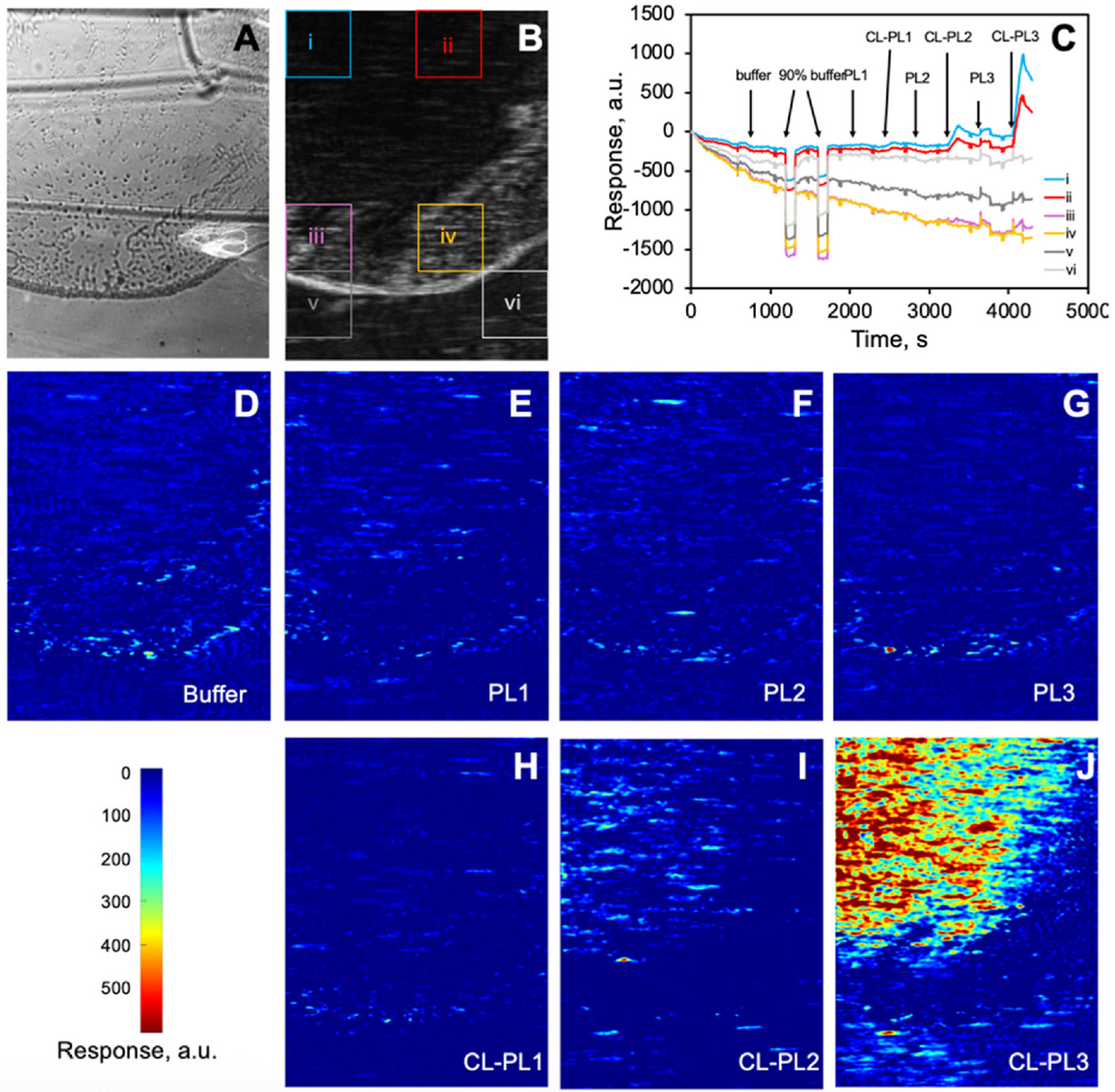
Representative surface plasmon resonance microscopy (SPRm) results showing liposome interactions with immobilized bacteria. (A) Bright-field image and (B) corresponding SPRm image highlighting the boundary of the bacterial deposition region. (C) Real-time SPRm signals extracted from six regions of interest (ROIs) indicated in (B). (D-J) SPRm images at the end of each injection after subtracting the corresponding first frame. All images are 600 μm × 450 μm in size.

We tested the interaction between varying concentrations of PL liposomes (PL1-3) and CL-PL liposome (CL-PL1-3) with SE using SPRm. Fig. 2A and B shows the correlated bright field and SPRm images from a SE deposited sensor chip, respectively. The SE deposition covers approximately ¾ of the sensor chip with a clear boundary. Both images indicate a higher density of SE near the boundary and a lower one inside the deposited spot. Moreover, there are spots observed outside the deposited spot (towards the bottom of the view), which could be impurities or some isolated SE.

During the SPRm flow cell test, a PBS-BSA buffer was passed through the system continuously with a series of injections as shown in the real-time sensorgrams in Fig. 2C. These sensorgrams were extracted from the six different regions of interests (ROIs, i - vi) as labelled in Fig. 2B, with ROIs i and ii covering the lower density SE inside the deposition spot, iii and iv covering the higher density SE at the boundary, and v and vi covering the background regions. With blank injection, since the same running buffer was injected, minimal signal change should be recorded, while for the calibration injection with 90% buffer, a sharp decrease in signal due to bulk refractive index change, followed by a short plateau period and then a sharp recovering of signal due to solution switching back to 100% buffer should be recorded. All regions showed similar response towards these two injections, as expected. The 90% buffer injection was used to calibrate signal changes from all ROIs to the same level for the following data analysis.

Upon liposome injections (PL1, CL-PL1, PL2, CL-PL2, PL3, CL-PL3), differences started to show among different ROIs. PL1 injection resulted in minimal signal changes at all ROIs, while CL-PL1 injection resulted in a small signal increase in ROIs i and ii, but not the others. This variation became much more apparent during the CL-PL2 and CL-PL3 injections. During both injection, ROIs i and ii showed a typical association profile during the exposing stage and a dissociation profile during the buffer washing stage, suggesting a strong kinetics interaction between the liposomes and the immobilized SE. ROIs iii and iv showed minimal signal changes, likely due to the boundary SE layer being too thick for the SPR sensing depth. The overall baseline drift in ROIs iii and iv also suggests that the SE group were less stable here due to high packing. PL2 injection generated minimal signal changes to all ROIs, while PL3 generated a small level of signal increase. That small change was mostly due to solution refractive index change other than association/dissociation since the profile followed the sharp increase-plateausharp decrease pattern. The difference between PL and CL-PL injections reveals that the strong interaction at ROIs i and ii originated from the CL component instead of PL.

Fig. 2D-J presents the distribution of liposome accumulation across the imaging area. Consistent with the real-time observation, buffer and PL injections caused minimal net signal change at the end, indicating no liposome accumulation. In contrast, CL-PL injections established a concentration dependence on the final liposome accumulation. The outside-deposition regions showed much less accumulation compared to inside regions, but there are individual spots which also showed concentration dependence, suggesting that these might be isolated SE spots. Overall, this capability of visualizing heterogeneity within a single sample of SPRm is beyond what can be achieved with traditional SPR spectroscopy.

**Fig. 3.**
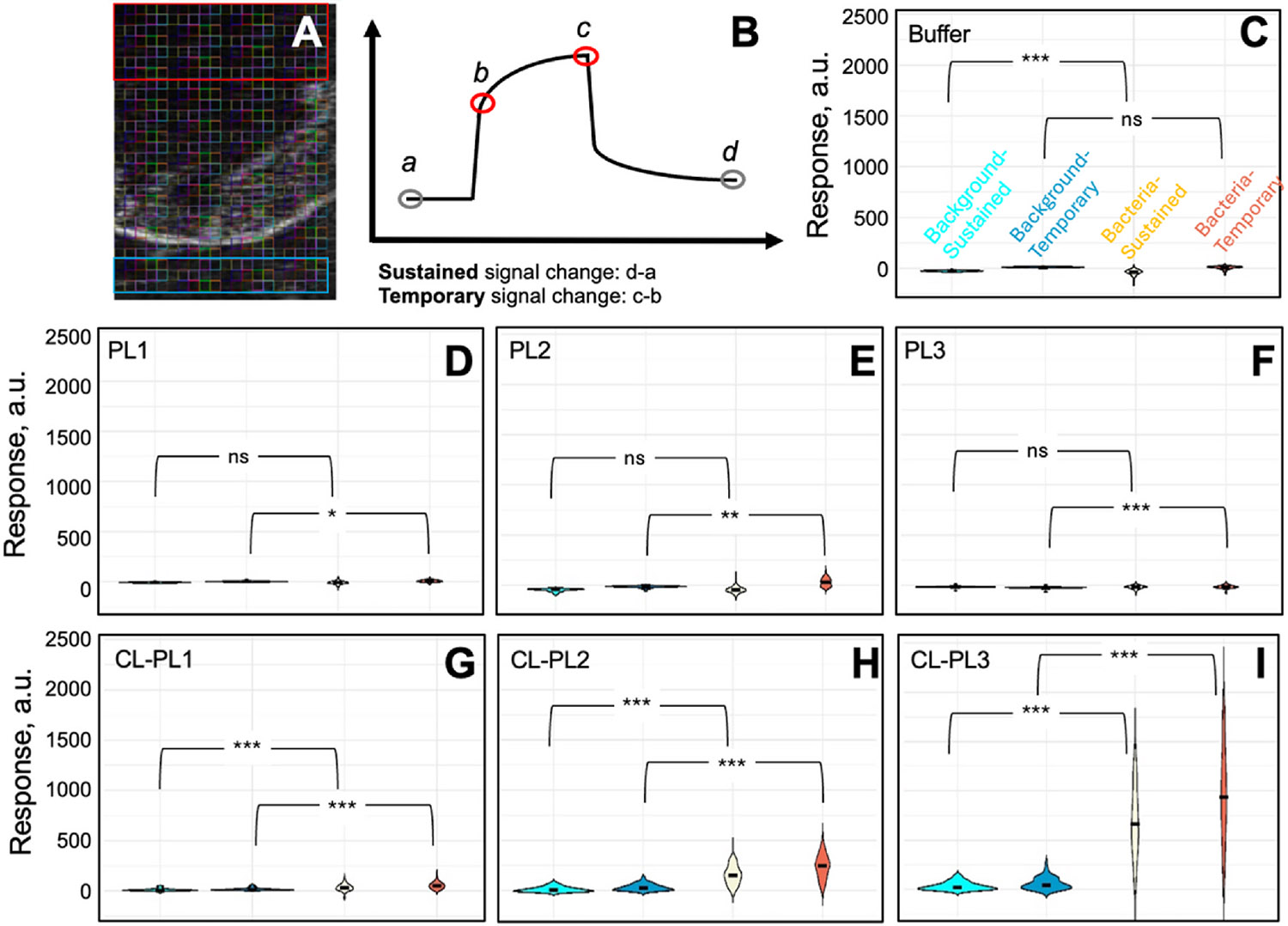
Quantitative comparison of the intensity changes among injections from Fig. 2. (A) SPRm image highlighting the square shaped ROIs across the sample. The red and blue rectangular shapes label the 140 ROIs included for bacteria and 60 for background regions, respectively. (B) Illustrates the selection of data points for each injection and the subsequent calculation to obtain two intensity change values, temporary and sustained. (C-I) The violin plots from all injections. The width of the violins indicates the population density at a given response unit, while the black bar in the center represents the median response. ns: not significant. *: p < 0.05. **: p < 0.01. ***p < 0.001 (see Tables S5 and S6).

To quantitatively and statistically compare signals from bacteria covered regions and background regions, we next divided the imaging area to square ROIs with each ROI covering approximately 21.8 μm by 21.8 μm (Fig. 3A) and extracted temporary and sustained signal changes as shown in Fig. 1A and 3B. Of note, in areas with bacteria, one ROI typically covers 100 – 400 individual bacteria (see Fig. 1F), thus the data shown in this figure represent over 20,000 individual bacteria. When analyzing all response units collected for this experiment, there was a statistically significant increase from bacteria area compared to background area (p < 0.001, see Tables S1 and S2 and Fig. S4). This strongly supports that this SPRm protocol captures true bacterial responses. When analyzing all response units from bacteria only, there was a significant difference between CL-PL and the other two treatment groups (p < 0.001, see Tables S3 and S4 and Fig. S5).

Fig. 3D-I shows violin plots of “Temporary” and “Sustained” values from both bacteria and background areas. Consistent with the visual observations in Fig. 2, only CL-PL injections generated higher “Temporary” and “Sustained” values and established a dose dependence at the bacteria regions than at the background regions. Moreover, in addition to the higher mean value, the bacteria regions also established a wider distribution in signal intensity, highlighting the heterogeneity within the bacteria group. The background regions generally gave around zero signals, except in CL-PL2 and CL-PL3 injections. At these two higher concentrations, there are apparently some ROIs establishing positive values, which are consistent with the visual observations that some isolated bacteria might be present outside the deposition spot.

One-Way ANOVA with Bonferroni post-hoc analysis revealed that the observed differences were statistically significant for PL for “Temporary” association between background and bacteria at all three concentrations and for CL-PL for both “Sustained” and “Temporary” association at all three concentrations (see Tables S5-S6). A statistically significant dose-dependency was verified for the three CL-PL injections but not for the PL injections (see Tables S7 and S8 and Fig. S6AB). Overall, these results indicate a strong dose-dependent interaction between CL-PL liposomes and SE during association stage and accumulation of CL-PL liposomes on the bacteria’s surface and an interaction between PL and SE to a much lesser degree.

**Fig. 4.**
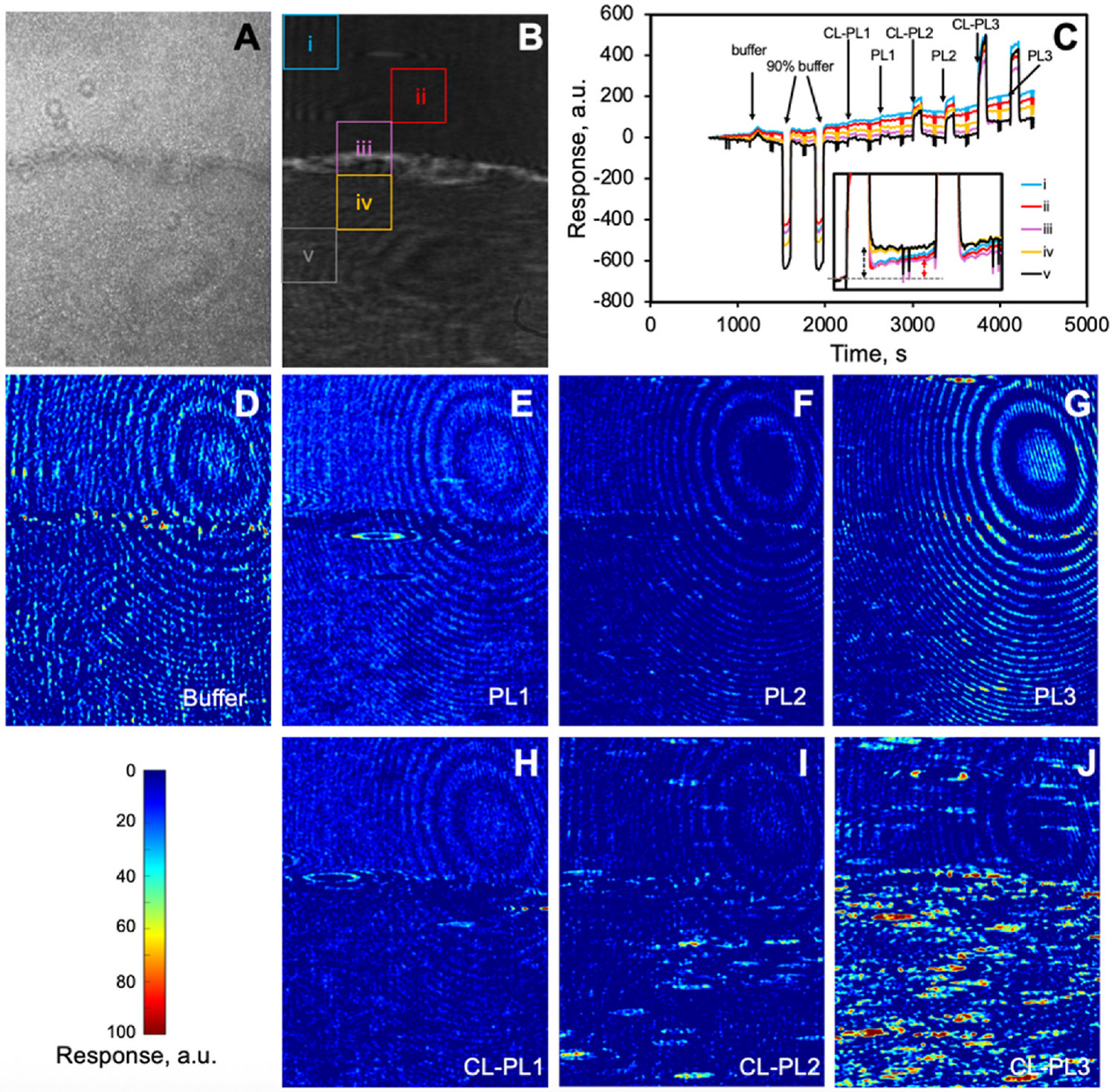
Representative SPRm results showing liposome interactions with immobilized bacteria. (A) Bright-field image and (B) corresponding SPRm image highlighting the boundary of the bacterial deposition region. (C) Real-time SPRm signals extracted from five regions of interest (ROIs) indicated in (B). (D-J) SPRm images at the end of each injection after subtracting the corresponding first frame. All images are 600 μm × 450 μm in size.

**Fig. 5.**
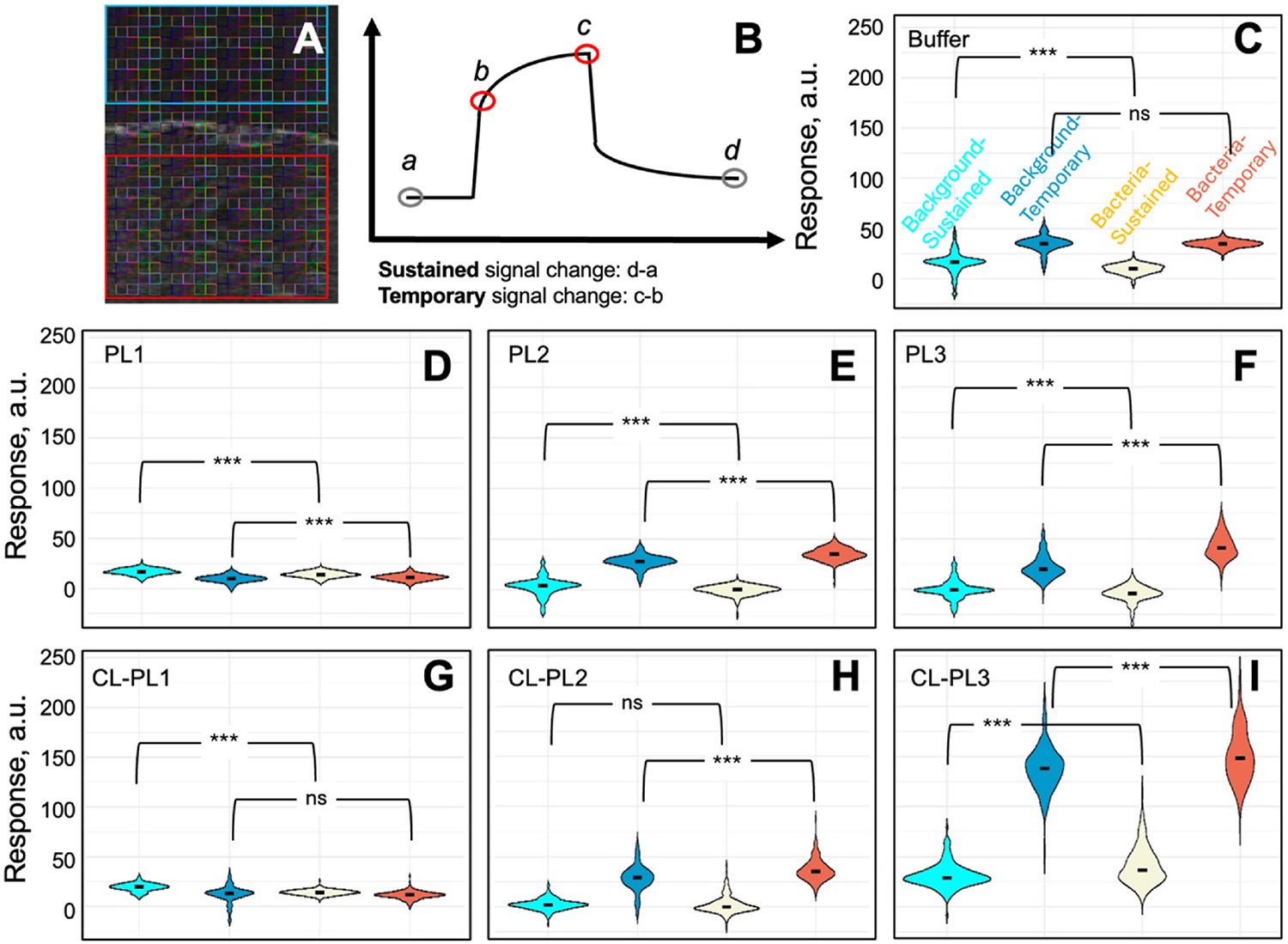
Quantitative comparison of the intensity changes among injections from Fig. 5. (A) SPRm image highlighting the square shaped ROIs across the sample. The red and blue rectangular shapes label the 260 ROIs included for bacteria and 180 for background regions, respectively. (B) Illustrates the selection of data points for each injection and the subsequent calculation to obtain two intensity change values, temporary and sustained. (C-I) The violin plots from all injections. ns: not significant. ***p < 0.001, (see Tables S13 and S14).

After establishing the above testing and data analysis protocols, we proceeded with testing a different sample with a reversed injection order (Figs. 4 and 5; CL-PL1, PL1, CL-PL2, PL2, CL-PL3, PL3). The difference between bacteria regions and background regions in real-time sensorgrams (Fig. 4C) was not as obvious as in Fig. 2C. Zoom-ins of the CL-PL3 and PL3 injections in Fig. 4C show that ROIs iv and v (bacteria, black and orange) established higher “Sustained” change (indicated by the black dashed arrow) compared to other ROIs (indicated by the red dashed arrow) during the CL-PL3 injection. In contrast, none of the ROIs showed visible “Sustained” change during the PL3 injection, which is consistent with the observation in the previous sample.

The SPRm images at the end of each injection are shown in Fig. 4D-J. There is a pattern with concentric rings of alternating bright and dark regions in the background region present in all the images due to a small air bubble appearing within the refractive index matching fluid that caused optical interference. This pattern is especially strong in the buffer injection (Fig. 4D). Apart from this noise pattern, the SPR signal itself shows minimal spatial variation. This observation applies to all PL injections as well (Fig. 4E-G). As for the CL-PL injections, the noise patten was less obvious, and more spots with positive “Sustained” signal change indicating accumulation appeared when the concentration of CL-PL increased (Fig. 4H-J). The overall signal change was less intense compared to the previous sample, likely due to a lower density of bacteria coverage.

When analyzing all response units collected for this experiment, there was a small but statistically significant increase in ROIs derived from bacteria compared to the ROIs derived from background area (p < 0.001, see Tables S9 and S10 and Fig. S7). When analyzing all response units from bacteria only, there was a significant difference between the three treatment groups overall (buffer, PL, and CL-PL) with CL-PL producing the highest response units (p < 0.001, see Tables S11 and S12 and Fig. S8).

The corresponding violin plots for this experiment (Fig. 5) show much lower signals compared to the previous sample. The data range in Fig. 5C-I (up to 250) is 10-times narrower than those in Fig. 3C-I (up to 2500), and the “Temporary” signals during buffer and PL injections are visually very apparent. In addition, the existence of a relatively high noise pattern (see Fig. 4) further obscures the interaction signals. Regardless of these factors, the CL-PL injection at its highest concentration (CL-PL 3, Fig. 5I) generated notable differences between bacteria and background regions. For the “Temporary” signals during CL-PL2 injection, although both background (dark blue) and bacteria (orange) regions established a positive mean value similar to the PL2 injection, there is a wider distribution in signal at the bacteria region, indicated by the top of the violin plot extending to about 100 a.u. Similarly, the “Sustained” signals from both background (light blue) and bacteria (beige) regions established mean values around 0, but the bacteria region has a small portion of violin plot extending to about 50 a.u. This is consistent with the SPRm image with a few “hotspots” within the bacteria region (Fig. 4I). With CL-PL3 injection, the difference between the background and bacteria regions was more apparent: both the “Temporary” and “Sustained” violin plots from bacteria regions established a wider distribution of signals towards the positive side, especially with the “Sustained” change. Overall, the weaker association/accumulation signal can be attributed to the lower bacteria coverage, and the slightly higher signal change during the PL injections could be contributed by the change of injection order. Specifically, the existence of CL-PL liposomes on the bacteria surface prior to each PL injection might have facilitated the temporary association of PL liposomes. One-way ANOVA revealed that only for CL-PL3 consistently significantly higher response units were observed for “Temporary” and “Sustained” association with bacteria (p < 0.001 across all other groups, Tables S13-S16 and Fig. S9AB).

Taken together, in both experiments, significant strong interaction between CL-PL and SE and some though much lesser and not always significant interaction between PL and SE was observed. This is in line with our previous data showing that PL liposomes effected some growth inhibition, but to a much lesser degree than CL-PL liposomes (Cheung Lam et al., 2016). For example, when employing a modified minimal inhibitory concentration assay, PL affected a 50% growth inhibition of SE while the CL containing formulation effected a complete growth inhibition. The SPRm visualizations highlighted population-level differences and heterogeneity in “Temporary” and “Sustained” association across the bacteria population reflected by the elongated distribution of signals. This was observed in both experiments, especially during the CL-PL3 injection. In our experiments, SE was harvested after 20 h of growth in TSB. This places the bacteria in the stationary growth phase, when bacteria are known to exhibit heterogeneity, with a mixture of live, dead, or metabolically inactive cells (Jaishankar and Srivastava, 2017) and differential gene expression (Vandecasteele et al., 2001). Thus, our data suggest that SPRm can capture bacterial heterogeneity within a population, which can be in particular useful for examining antimicrobial drug resistance (Huemer et al., 2020; Pani and Mohapatra, 2024).

### 3.3. Kinetic analysis

**Fig. 6.**
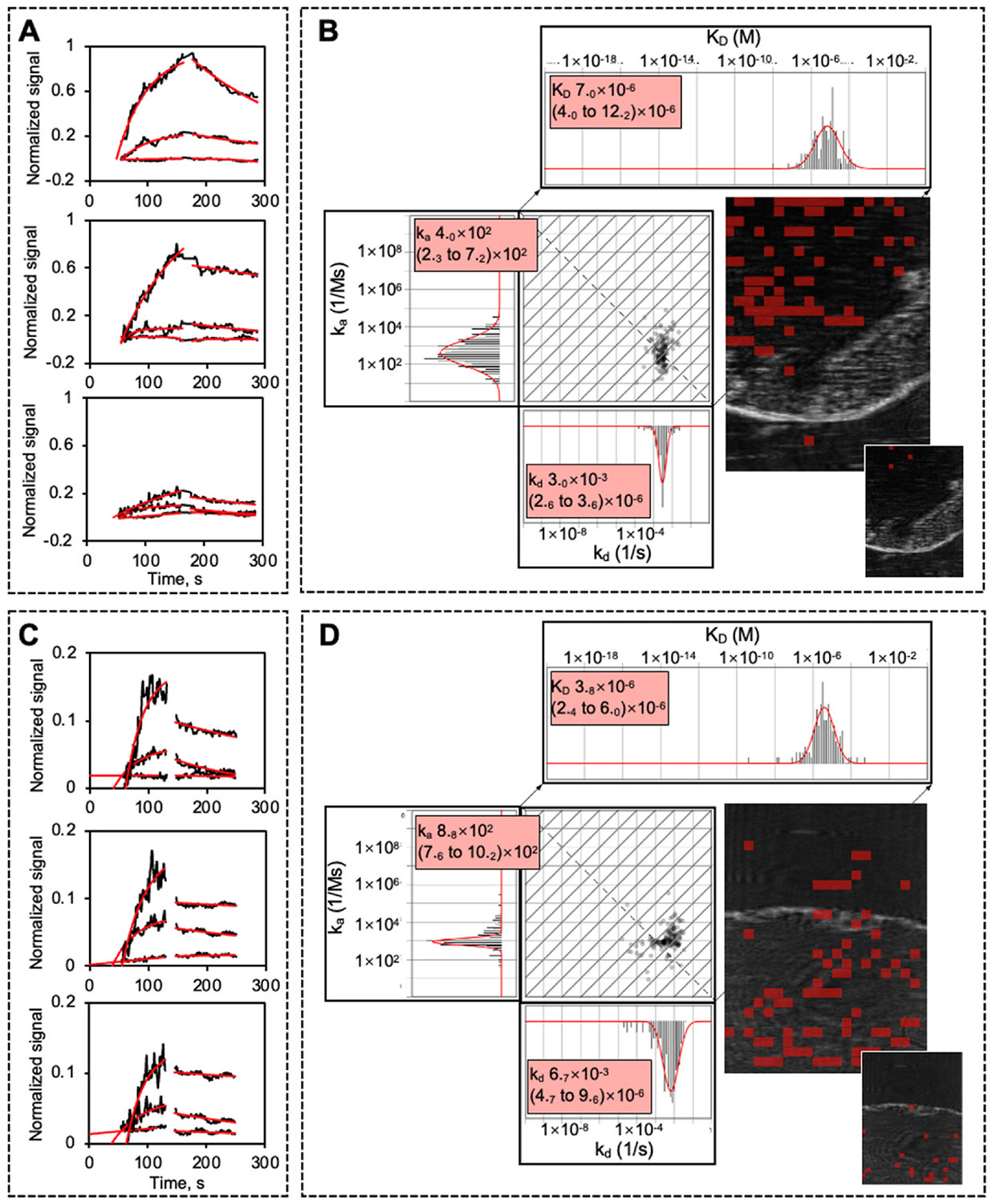
Kinetic analysis of bacteria-liposome interactions based on data from (A, B) Fig. 2 and (C, D) Fig. 4. (A, C) Representative sensorgrams extracted from individual ROIs (black) overlaid with fits using a 1:1 Langmuir kinetic model (red). (B, D) Isoaffinity scatter plots illustrating the kinetic heterogeneity of the binding events. Histograms for the association rate constant (k_a_), dissociation rate constant (k_d_), and equilibrium dissociation constant (K_D_) are fitted with Gaussian distributions to determine mean values and 95% confident intervals (CI). The inset SPRm images highlight ROIs that exhibited high-quality kinetic fits; larger images represent CL-PL injections, while smaller thumbnails represent PL injections.

After evaluating the overall liposome accumulation at individual ROIs, we explored the underlying interaction kinetics by fitting the association and dissociation profiles to a 1:1 Langmuir binding model. Representative sensorgrams extracted from randomly selected ROIs (black lines, Fig. 6A and C) were directly overlaid with the theoretical fits (red lines), providing a clear visual confirmation of the model’s suitability for our experiments. After eliminating the outlier ROIs that yielded invalid kinetic fits, the vast majority of remaining valid ROIs for the CL-PL injections localize specifically within the bacteria area (Fig. 6B and D), demonstrating that the interaction is highly bacteria-specific. In contrast, fitting the data from the PL-only injections yielded minimal valid ROIs (smaller thumbnails in Fig. 6B and D, further confirming that the observed binding is driven by CL.

Despite the differences in signal magnitude between the two experimental runs, the extracted kinetic parameters for the valid ROIs in both remain within the same order of magnitude. A major advantage of this spatially resolved technique over traditional bulk assays is its capacity to reveal the significant heterogeneity of these interactions across different cellular regions, as evidenced by the broad distributions in the scatter plots. The observed micromolar-range K_D_ indicates a relatively weak interaction when compared to highly specific antibody-antigen binding, which typically exhibits nM affinities. Nevertheless, a key limitation of this analysis is its reliance on a strict 1:1 kinetic assumption; the actual physical interactions are likely multivalent and considerably more complex.

## 4. Conclusions

For the first time, we established SPRm to investigate the real-time interaction between antimicrobial liposomes and live SE. The imaging capability of SPRm enabled spatially resolved detection of the association/dissociation kinetics across the sample, which allows to monitor the difference between well-defined bacteria covered regions and background regions as well as the heterogeneity within the bacteria population. We successfully demonstrated the accumulation of CL-PL liposomes specifically on the SE surface. A large variation in signals across the bacteria deposited region was also observed, indicating the heterogeneous nature of the interaction, which could not be possibly studied with conventional techniques. Additionally, a novel protocol involving placing the SPRm sensor chip onto an agar containing tetrazolium salt was successfully developed to ensure that the SPRm procedure itself does not negatively impact the viability of the immobilized bacteria. For future work, the SPRm system can be combined with surface characterization techniques such as AFM to further dissect morphological changes of the bacteria surface. The system itself could be upgraded with additional detecting channels, such as electrochemical detection of reactive molecules generated during the process. Furthermore, using genetically modified bacteria may enable mechanistic studies of antibacterial drugs and host defense molecules. Overall, this work presents a novel approach of studying antimicrobial activities capable to discern heterogeneity within live bacterial populations.

## Supplementary Material

Supplementary

## Data Availability

Data used to generate figures for this manuscript can be accessed through Zenodo (https://doi.org/10.5281/zenodo.19501983) after publication.

## Appendix A. Supplementary data

Supplementary data to this article can be found online at https://doi.org/10.1016/j.biosx.2026.100792.
