# Supplementary material for: Accessing real-time interaction between antimicrobial liposomes and live Staphylococcus epidermidis using surface plasmon resonance microscopy
Source: Biosens Bioelectron X. Author manuscript; Available in PMC 2026 Jul 15. (PMC13364176; doi:10.1016/j.biosx.2026.100792)
Supplement: Supplementary [file NIHMS2193189-supplement-Supplementary.docx]

**Supplementary Material**

**Accessing real-time interaction between antimicrobial liposomes and live *Staphylococcus epidermidis* using surface plasmon resonance microscopy**

Kam Hang Chan^1^, Nancy Diaz Batista^2^, Kevin Diego-Perez^1^, Adaly Garcia^1^, Rachelle Soriano^1^, Maria Kala^2^, Juan Luis Cazares^2^, Sheehan Belleca^1^, Stellina Ao^1^, Christina Dhoj^1^, Zhipeng Dai^3^, Sam On Ho^3^, Gary Fujii^3^, Edith Porter^2,^*, Yixian Wang^1,^*

^1^Department of Chemistry and Biochemistry, California State University, Los Angeles, Los Angeles, CA 90032, USA

^2^Department of Biological Sciences, California State University, Los Angeles, Los Angeles, CA 90032, USA

^3^Molecular Express, Inc., Rancho Dominguez, CA 90220, USA

Corresponding authors

Edith Porter; [eporter@calstatela.edu](mailto:eporter@calstatela.edu)

Department of Biological Sciences, California State University, Los Angeles, Los Angeles, CA, USA

Yixian Wang; [ywang184@calstatela.edu](mailto:ywang184@calstatela.edu)

Department of Chemistry and Biochemistry, California State University, Los Angeles, Los Angeles. CA, United States

**Table of contents**

1. Liposome preparation---------------------------------------------------------------------------------------S4
2. Bacteria culture----------------------------------------------------------------------------------------------S4
3. Quantifying the binding interactions----------------------------------------------------------------------S4
4. Bacteria characterization------------------------------------------------------------------------------------S5

**Fig. S1.** Unsuccessful attempts of viability staining of SE.

**Fig. S2.** Motility agar with tetrazolium (Hardy Diagnostics) right after inoculating SE with a needle and after overnight incubation.

**Fig. S3.** Viability test for SE with tetrazolium agar.

1. Statistical analysis of signal changes for data from Figs. 2 and 3 ------------------------------------S7
   1. *Overall comparison between background and bacteria areas (data from Figs. 2 and 3).* ---S7

**Table S1.** Descriptive statistics of overall background and bacteria area signal changes including both, sustained and temporary response units (*data from* Figs. 2 and 3).

**Table S2.** Independent Samples T test for comparing overall background and bacteria area signal changes including both, sustained and temporary response units (*data from* Figs. 2 and 3).

**Fig. S4.** Overall comparison between the means of background and bacteria area signal changes including both, sustained and temporary response units (data from Figs. 2 and 3). Error bar stands for the standard error.

- 1. *Overall comparison between CL-PL, PL and buffer injections at bacteria area (data from Figs. 2 and 3).* ------------------------------------------------------------------------------------------S8

**Table S3.** Descriptive statistics of signal changes during CL-PL, PL, and buffer injections at bacteria area including both, sustained and temporary response units (data from Figs. 2 and 3).

**Table S4.** One-Way ANOVA results for CL-PL, PL, and buffer injections at bacteria area including both, sustained and temporary response units (data from Figs. 2 and 3).

**Fig. S5.** Overall comparison between the means of signal changes during CL-PL, PL and buffer injections including both, sustained and temporary response units (data from Figs. 2 and 3). Error bar stands for the standard error.

- 1. *Comparison between groups and injections (data from Figs. 2 and 3).* -------------------------S9

**Table S5.** Descriptive statistics of all groups at all injections (data from Figs. 2 and 3).

**Table S6.** One-Way ANOVA comparing background and bacteria signals for sustained and temporary responses at each injection (data from Figs. 2 and 3).

**Table S7.** One-Way ANOVA results for the sustained signal change from all injections at bacteria area (data from Figs. 2 and 3)

**Table S8.** One-Way ANOVA results of the temporary signal change from all injections at bacteria area (data from Figs. 2 and 3)

**Fig. S6.** Comparison between the means of (A) sustained and (B) temporary signal changes from all injections at bacteria area (data from Figs. 2 and 3). Error bar stands for the standard error.

1. Statistical analysis of signal changes for *data from Figs. 4 and 5* ----------------------------------S12
   1. *Overall comparison between background and bacteria areas (data from Figs. 4 and 5).*--S12

**Table S9.** Descriptive statistics of overall background and bacteria area signal changes including both, sustained and temporary response units (*data from* Figs. 4 and 5).

**Table S10.** Independent Samples T test for comparing overall background and bacteria area signal changes including both, sustained and temporary response units (*data from* Figs. 4 and 5).

**Fig. S7.** Overall comparison between the means of background and bacteria area signal changes including both, sustained and temporary response units (data from Figs. 4 and 5). Error bar stands for the standard error.

*6.2 Overall comparison between CL-PL, PL and buffer injections at bacteria area (data from Figs. 4 and 5).* ----------------------------------------------------------------------------------------------S13

**Table S11.** Descriptive statistics of overall signal changes during CL-PL, PL, and buffer injections at bacteria area including both, sustained and temporary response units (data from Figs. 4 and 5).

**Table S12.** One-Way ANOVA results for CL-PL, PL, and buffer injections at bacteria area including both, sustained and temporary response units (data from Figs. 4 and 5).

**Fig. S8.** Overall comparison between the means of signal changes during CL-PL, PL and buffer injections including both, sustained and temporary response units (data from Figs. 4 and 5). Error bar stands for the standard error.

- 1. *Comparison between groups and injections (data from Figs. 4 and 5)----------*---------------S14

**Table S13.** Descriptive statistics of all groups at all injections (data from Figs. 4 and 5).

**Table S14.** One-Way ANOVA comparing background and bacteria signals for sustained and temporary responses at each injection (data from Figs. 4 and 5).

**Table S15.** One-Way ANOVA results for the sustained signal change from all injections at bacteria area (data from Figs. 4 and 5).

**Table S16.** One-Way ANOVA results of the temporary signal change from all injections at bacteria area (data from Figs. 4 and 5).

**Fig. S9.** Comparison between the means of (A) sustained and (B) temporary signal changes from all injections at bacteria area (data from Figs. 4 and 5). Error bar stands for the standard error.

1. MATLAB code for SPRm image processing------------------------------------------------------------S17
2. R code for making violin plots ---------------------------------------------------------------------------S20

# **1. Liposome preparation**

Hydrogenated soy phosphatidylcholine (HSPC) and distearoyl-phosphatidylglycerol (DSPG) were bought from Avanti Polar Lipids, Inc. (Alabaster, AL). CL was purchased from Sigma-Aldrich. Lyophilized lipids were dissolved separately in chloroform:ethanol 3:1 (v:v) solution to obtain working stocks of CL (MW 649.1, 5 mg/mL), HSPC (MW 783.77, 10 mg/mL), and DSPG (MW 801.06, 1 mg/mL), whereby 250 μL of HPLC grade dH_2_O of water was added to DSPG, after the addition of the organic solvent. Then, the lipid solutions were mixed in 16 × 100 mm borosilicate glass tubes to yield the desired lipid quantities, followed by flushing with N_2_ gas, which was performed to evaporate the solvent, while preventing oxidation of fatty acids, and desiccation under negative vacuum pressure (<5 microns Hg) for 4 days. Dry lipid films were rehydrated in aqueous phase with the addition of 10 mL 100 mM sodium phosphate, pH 7.04 (pre-warmed to 45°C) for 10 min, followed by probe-sonication (U.S.Solid, 450W ultrasonic homogenizer, with 3 mm probe, at 25 % power over 5 min (with 10 second on, followed by 5 second off) in 45°C water bath) and sterile filtration through a 0.22 µm pore size filter (Millex-GP filter with polyethersulfone membrane, Millipore Sigma, Burlington, MA, USA).

**2. Bacteria culture**

When needed, one cryobead (prepared according to the manufactirer’s recommendations (Hardy Diagnostics) and kept at -80°C) was streaked onto a TSA plate and the plate was incubated for 24 h at 37 °C. From this, one isolated colony was inoculated into 5 mL of TSB and incubated for 20 h at 37 °C to yield stationary-phase bacteria. Then, 1 mL of the culture was spun down for 2 min @ 12,000 rpm in a tabletop centrifuge. The pellet was washed twice with 1 mL of dH_2_O for 2 min @ 12,000 rpm and then resuspended in 1 mL of dH_2_O (yielding approximately 1 x 10^9^ CFU/mL) and further diluted in 1:2 in dH_2_O or as needed and kept on ice for up to 30 min until further experimentation. Water was used to wash and resuspend the bacteria to avoid deposition of salts that would interfere with imaging. As Gram-positive bacteria with a thick cell wall, SE can be washed and temporarily maintained in water.

**3. Quantifying the binding interactions.**

As shown in Fig. 1 theoretical sensorgrams, point *a* represents the starting of the recording. Once the injection starts, a sharp increase in signal originates from the bulk refractive index change, which is established quickly before Point *b*. The association-caused signal change dominates the profile between points *b* and *c*. Starting from point *c*, the buffer rinsing generates a sharp signal decrease again due to refractive index changing back to running buffer, followed by a gradual signal decrease dominated by dissociation till the end point of recording, point *d*. Two quantities were extracted from each intensity profiles: the signal increase from point *b* to point *c*, which represents the temporary liposome accumulation, and the signal increase from point *a* to point *d*, which represents the sustained liposome accumulation. The exact timing of points *b* and *c* can be determined using the calibration injection since it does not involve the association/dissociation factor. The data were also categorized into bacterial and background areas based on the microscopic images of the chip.

# **4. Bacteria characterization**


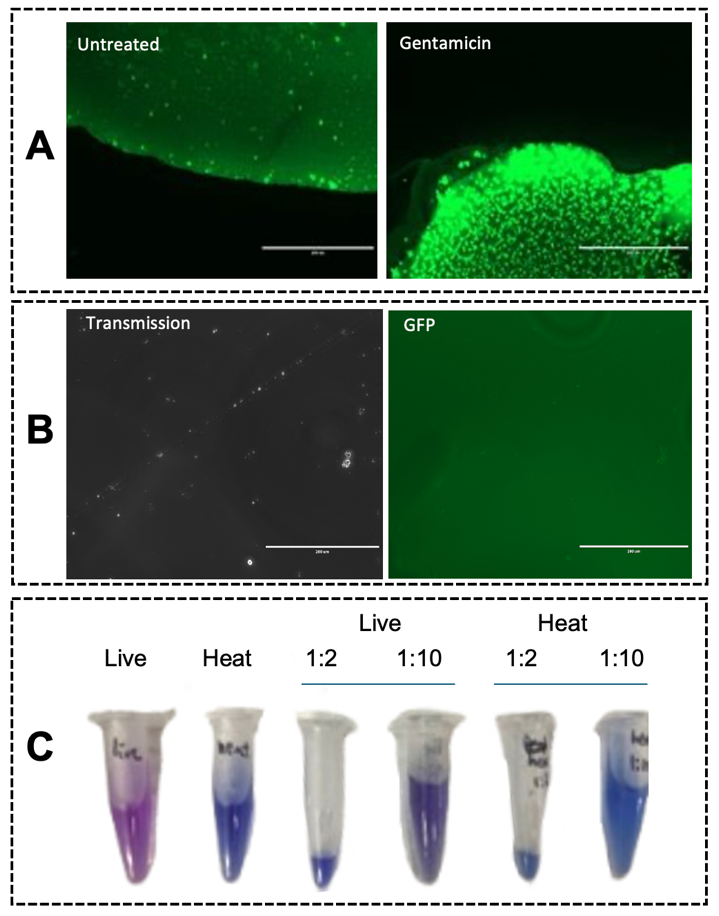


**Fig. S1.** Unsuccessful attempts of viability staining of SE. A. Fluorescence staining using BCECF-AM (Molecular Probes, 2 mM, 30 min at 37°C) with prominent green fluorescence of gentamicin treated (1 mg/mL for 30 min at 37°C) bacteria that exceed the green fluorescence of untreated live SE. Scale bar: 200mm. B. Absence of green fluorescence in live untreated bacteria with Calcein-AM (Molecular Probes, 250 nM, 30 min at 37°C). Scale bar: 200 mm. C. Resazurin (Sigma Aldrich, incubation with 0.01% for 1 h at 37°C) is not sensitive enough to differentiate live and heat killed SE at concentrations used for SPRm. The pink color that reflects metabolic activity of live SE leading to the reduction of the blue resazurin to the pink, is lost after further dilution. For all attempts bacteria were grown for 20 h at 37°C in TSB then washed and resuspended in equal volume of dH_2_O.

**
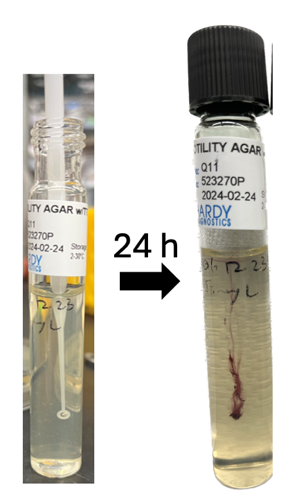
**

**Fig. S2.** Motility agar with tetrazolium (Hardy Diagnostics) right after inoculating SE with a needle and after overnight incubation. Motility agar media are formulated at a lower agar concentration to yield a semi solid agar. Once inoculated with a needle two thirds into the agar, motile bacteria are able to disseminate throughout the agar while non motile bacteria will be arrested at the inoculation site and proliferate only along the inoculation. Live bacteria reduce the colorless tetrazolium salt into red formazan, producing a prominent color change that indicates viability. Thus, this medium allows for the differentiation of motile bacteria showing diffuse red color change throughout the medium from non-motile bacteria showing red color only at the site of inoculation.


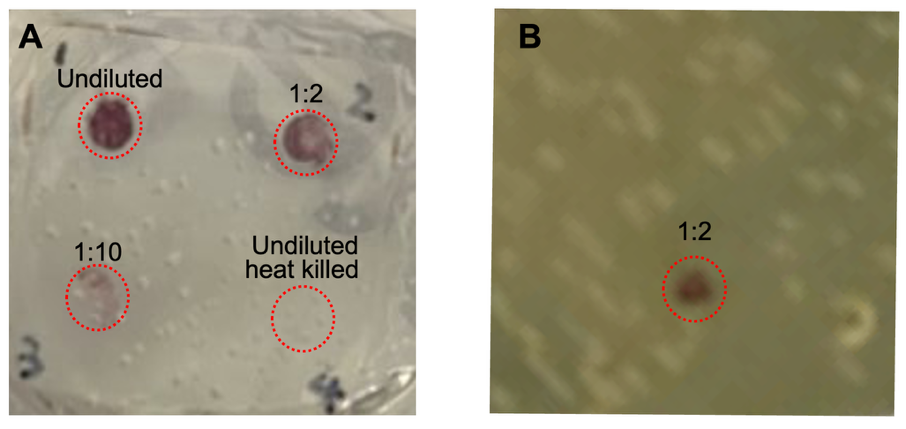


**Fig. S3.** Viability test for SE with tetrazolium agar. (A) Live bacteria at different concentrations (undiluted: ~ 1 × 10^9^ CFU/mL or 2 × 10^6^ bacteria in 2 μL, 1:2 dilution, and 1:10 dilution) as well as undiluted heat-killed (dead) bacteria were deposited onto a coverslip (2 μL per spot) according to the SPRm imaging protocol and immediately after drying the coverslip was placed onto the tetrazolium agar, bacteria facing the agar. As expected, heat-killed SE exhibited no reduction of the tetrazolium as no color developed, where the other three spots with live SE all developed red color that was inoculum dependent. (B) Live bacteria at a 1:2 dilution were spotted onto a modified sensor chip and after a SPRm flow test with running buffer only the chip was placed onto tetrazolium agar. Images were taken after 24 h incubation at 37°C. The original spot where the bacteria were deposited appears as a red spot comparable to the spot produced by the 1:2 diluted bacteria shown in A.

# **5. Statistical analysis of signal changes for data from Figs. 2 and 3**

- 1. *Overall comparison between background and bacteria areas (data from Figs. 2 and 3).,*

**Table S1.** Descriptive statistics of overall background and bacteria area signal changes including both, sustained and temporary response units (data from Figs. 2 and 3).

| **Groups** | **N** | **Mean** | **Std. Deviation** | **Std. Error** |
| --- | --- | --- | --- | --- |
| Background | 840 | 6.8604 | 33.70867 | 1.16306 |
| Bacteria | 1960 | 152.3814 | 337.05167 | 7.61322 |

**Table S2.** Independent Samples T test for comparing overall background and bacteria area signal changes including both, sustained and temporary response units (data from Figs. 2 and 3).

| ***p* value** | Background | Bacteria |
| --- | --- | --- |
| Background |  | <0.001 |
| Bacteria | <0.001 |  |

**
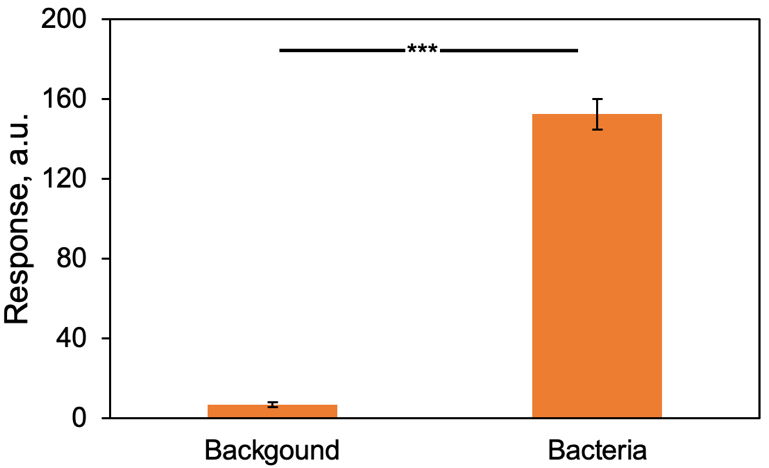
**

**Fig. S4.** Overall comparison between the means of background and bacteria area signal changes including both, sustained and temporary response units (data from Figs. 2 and 3). Error bar stands for the standard error.

- 1. *Overall comparison between CL-PL, PL and buffer injections at bacteria area (data from Figs. 2 and 3).*

**Table S3.** Descriptive statistics of overall signal changes during CL-PL, PL, and buffer injections at bacteria area including both sustained and temporary response units (data from Figs. 2 and 3).

| **Groups** | **N** | **Mean** | **Std. Deviation** | **Std. Error** |
| --- | --- | --- | --- | --- |
| Buffer | 280 | -14.2857 | 33.37082 | 1.99429 |
| PL | 840 | -.1122 | 32.12100 | 1.10828 |
| CL-PL | 840 | 360.4307 | 433.55002 | 14.95890 |

**Table S4.** One-Way ANOVA results for CL-PL, PL, and buffer injections at bacteria area including both, sustained and temporary response units (data from Figs. 2 and 3).

| ***p* value** | Buffer | PL | CL-PL |
| --- | --- | --- | --- |
| Buffer |  | 1.000 | <0.001 |
| PL | 1.000 |  | <0.001 |
| CL-PL | <0.001 | <0.001 |  |


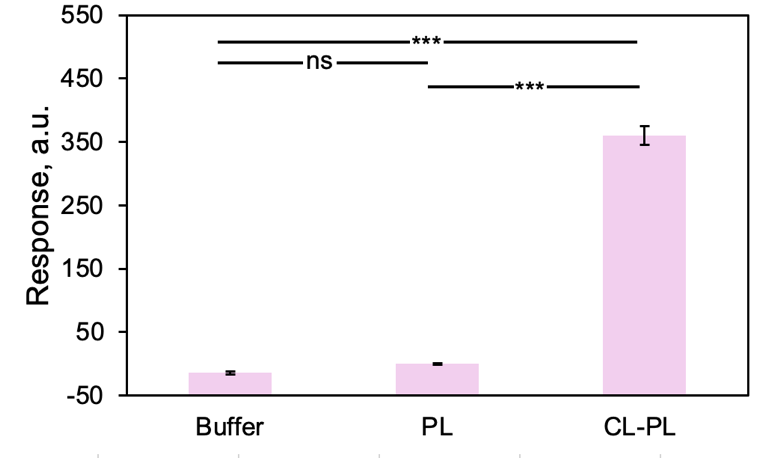


**Fig. S5.** Overall comparison between the means of signal changes including both, sustained and temporary response units during CL-PL, PL and buffer injections (data from Figs. 2 and 3). Error bar stands for the standard error.

- 1. *Comparison between groups and injections (data from Figs. 2 and 3).*

**Table S5.** Descriptive statistics of all groups at all injections (data from Figs. 2 and 3).

| **Injection** | **Groups** | **N** | **Mean** | **Std. Deviation** | **Std. Error** |
| --- | --- | --- | --- | --- | --- |
| Buffer | Background-Sustained | 60 | -24.6342 | 6.21243 | 0.80202 |
|  | Background-Temporary | 60 | 12.7951 | 4.09989 | 0.52929 |
|  | Bacteria-Sustained | 140 | -37.5579 | 29.26181 | 2.47307 |
|  | Bacteria-Temporary | 140 | 8.9865 | 16.97053 | 1.43427 |
| PL1 | Background-Sustained | 60 | -9.3081 | 4.05124 | 0.52301 |
|  | Background-Temporary | 60 | 0.4375 | 4.2487 | 0.5485 |
|  | Bacteria-Sustained | 140 | -9.1637 | 18.09164 | 1.52902 |
|  | Bacteria-Temporary | 140 | 5.6786 | 11.13695 | 0.94124 |
| PL2 | Background-Sustained | 60 | -14.7531 | 7.38703 | 0.95366 |
|  | Background-Temporary | 60 | -21.9332 | 7.37346 | 0.95191 |
|  | Bacteria-Sustained | 140 | -17.8808 | 19.56141 | 1.65324 |
|  | Bacteria-Temporary | 140 | -14.0610 | 17.50524 | 1.47946 |
| PL3 | Background-Sustained | 60 | -9.3730 | 14.53525 | 1.87649 |
|  | Background-Temporary | 60 | -7.9520 | 10.85095 | 1.40085 |
|  | Bacteria-Sustained | 140 | -4.3682 | 33.91124 | 2.86602 |
|  | Bacteria-Temporary | 140 | 39.1220 | 41.69381 | 3.52377 |
| CL-PL1 | Background-Sustained | 60 | 11.2463 | 12.26311 | 1.58316 |
|  | Background-Temporary | 60 | 18.4411 | 12.91344 | 1.66712 |
|  | Bacteria-Sustained | 140 | 36.7494 | 34.15955 | 2.88701 |
|  | Bacteria-Temporary | 140 | 56.0055 | 33.99836 | 2.87339 |
| CL-PL2 | Background-Sustained | 60 | 11.0942 | 25.48319 | 3.28987 |
|  | Background-Temporary | 60 | 31.6752 | 33.15349 | 4.28010 |
|  | Bacteria-Sustained | 140 | 156.8219 | 96.09144 | 8.12121 |
|  | Bacteria-Temporary | 140 | 242.3890 | 120.72405 | 10.20304 |
| CL-PL3 | Background-Sustained | 60 | 36.1332 | 41.13649 | 5.31070 |
|  | Background-Temporary | 60 | 62.1762 | 62.66844 | 8.09046 |
|  | Bacteria-Sustained | 140 | 701.6765 | 367.23877 | 31.03734 |
|  | Bacteria-Temporary | 140 | 968.9418 | 478.19501 | 40.41486 |

**Table S6.** One-Way ANOVA comparing background and bacteria signals for sustained and temporary responses at each injection (data from Figs. 2 and 3).

| **Injection** | **Background vs Bacteria** | ***p* value** |
| --- | --- | --- |
| Buffer | Sustained | <0.001 |
|  | Temporary | 1.000 |
| PL1 | Sustained | 1.000 |
|  | Temporary | 0.049 |
| PL2 | Sustained | 1.000 |
|  | Temporary | 0.010 |
| PL3 | Sustained | 1.000 |
|  | Temporary | <0.001 |
| CL-PL1 | Sustained | <0.001 |
|  | Temporary | <0.001 |
| CL-PL2 | Sustained | <0.001 |
|  | Temporary | <0.001 |
| CL-PL3 | Sustained | <0.001 |
|  | Temporary | <0.001 |

**Table S7.** One-Way ANOVA results for the sustained signal change from all injections at bacteria area (data from Figs. 2 and 3)

| ***p* value** | buffer | PL 1 | PL 2 | PL 3 | CL-PL 1 | CL-PL 2 | CL-PL 3 |
| --- | --- | --- | --- | --- | --- | --- | --- |
| buffer |  | 1.000 | 1.000 | 1.000 | <0.001 | <0.001 | <0.001 |
| PL1 | 1.000 |  | 1.000 | 1.000 | 0.176 | <0.001 | <0.001 |
| PL2 | 1.000 | 1.000 |  | 1.000 | 0.036 | <0.001 | <0.001 |
| PL3 | 1.000 | 1.000 | 1.000 |  | 0.382 | <0.001 | <0.001 |
| CL-PL1 | <0.001 | 0.176 | 0.036 | 0.382 |  | <0.001 | <0.001 |
| CL-PL2 | <0.001 | <0.001 | <0.001 | <0.001 | <0.001 |  | <0.001 |
| CL-PL3 | <0.001 | <0.001 | <0.001 | <0.001 | <0.001 | <0.001 |  |

**Table S8.** One-Way ANOVA results of the temporary signal change from all injections at bacteria area (data from Figs. 2 and 3)

| ***p* value** | buffer | PL 1 | PL 2 | PL 3 | CL-PL 1 | CL-PL 2 | CL-PL 3 |
| --- | --- | --- | --- | --- | --- | --- | --- |
| buffer |  | 1.000 | 1.000 | 1.000 | 0.765 | <0.001 | <0.001 |
| PL 1 | 1.000 |  | 1.000 | 1.000 | 0.529 | <0.001 | <0.001 |
| PL 2 | 1.000 | 1.000 |  | 0.378 | 0.039 | <0.001 | <0.001 |
| PL 3 | 1.000 | 1.000 | 0.378 |  | 1.000 | <0.001 | <0.001 |
| CL-PL 1 | 0.765 | 0.529 | 0.039 | 1.000 |  | <0.001 | <0.001 |
| CL-PL 2 | <0.001 | <0.001 | <0.001 | <0.001 | <0.001 |  | <0.001 |
| CL-PL 3 | <0.001 | <0.001 | <0.001 | <0.001 | <0.001 | <0.001 |  |


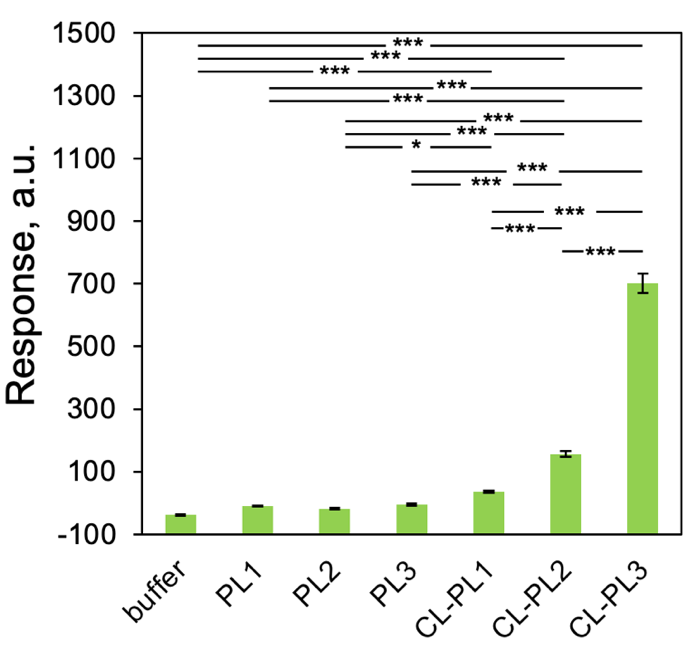

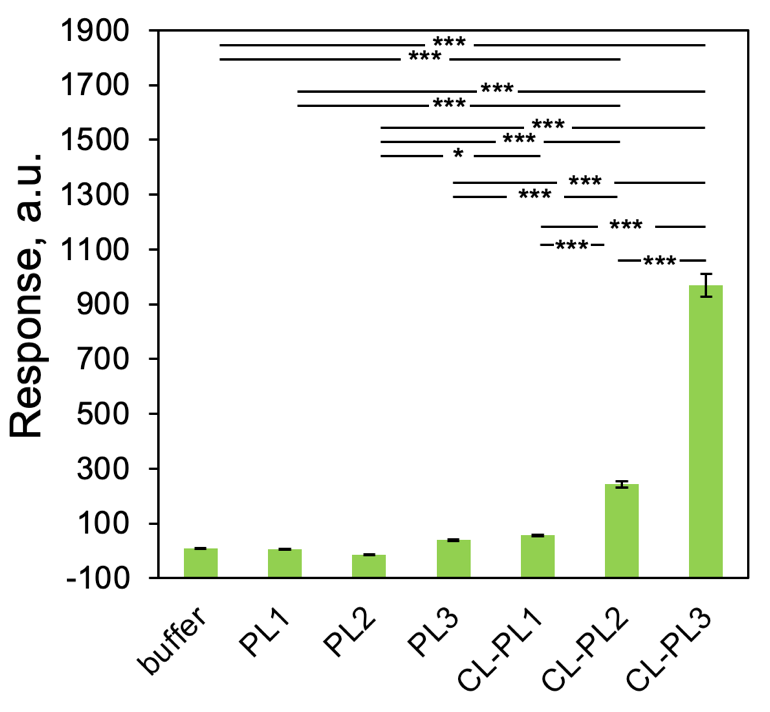


**A**

**B**

**Fig. S6.** Comparison between the means of (a) sustained and (b) temporary signal changes from all injections at bacteria area (data from Figs. 2 and 3). Error bar stands for the standard error.

# **Statistical analysis of signal changes *(data from Figs. 4 and 5)***

- 1. *Overall comparison between background and bacteria areas (data from Figs. 4 and 5).*

**Table S9.** Descriptive statistics of overall background and bacteria area signal changes including both, sustained and temporary response units (data from Figs. 4 and 5).

| **Groups** | **N** | **Mean** | **Std. Deviation** | **Std. Error** |
| --- | --- | --- | --- | --- |
| Background | 2520 | 25.9285 | 34.40079 | 0.68528 |
| Bacteria | 3640 | 28.5404 | 38.72369 | 0.64184 |

**Table S10.** Independent Samples T test for comparing overall background and bacteria area signal changes including both, sustained and temporary response units (data from Figs. 4 and 5).

| ***p* value** | Background | Bacteria |
| --- | --- | --- |
| Background |  | <0.001 |
| Bacteria | <0.001 |  |

**
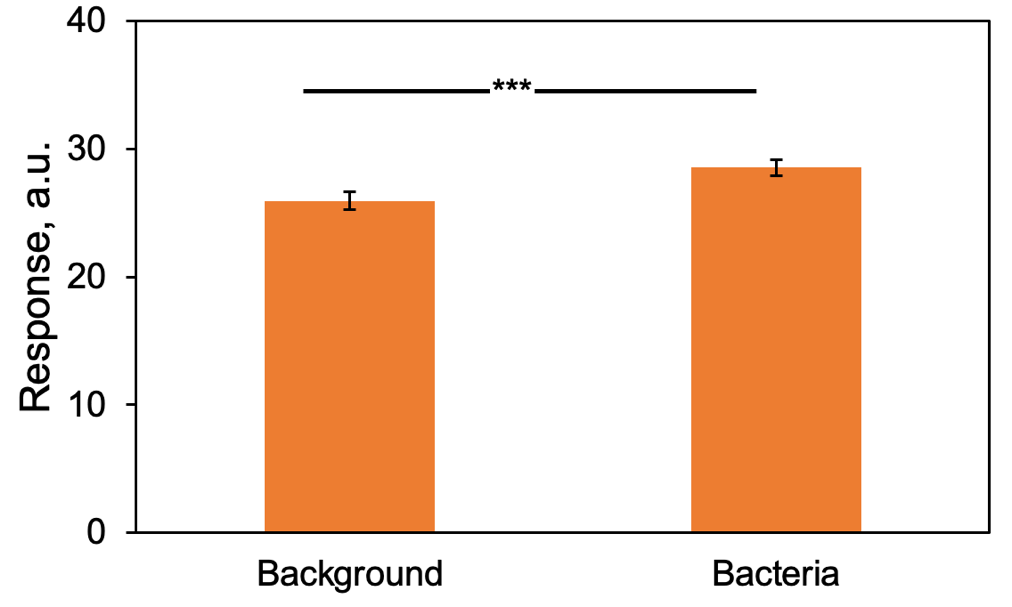
**

**Fig. S7.** Overall comparison between the means of background and bacteria area signal changes (data from Figs. 4 and 5). Error bar stands for the standard error.

- 1. *Overall comparison between CL-PL, PL and buffer injections at bacteria area (data from Figs. 4 and 5).*

**Table S11.** Descriptive statistics of overall signal changes including both, sustained and temporary response units during CL-PL, PL, and buffer injections at bacteria area (data from Figs. 4 and 5).

| **Groups** | **N** | **Mean** | **Std. Deviation** | **Std. Error** |
| --- | --- | --- | --- | --- |
| Buffer | 520 | 22.2771 | 13.07287 | .57328 |
| PL | 1560 | 16.3971 | 18.46003 | .46738 |
| CL-PL | 1560 | 42.7715 | 52.33748 | 1.32511 |

**Table S12.** One-Way ANOVA results for CL-PL, PL, and buffer injections at bacteria area (data from Figs. 4 and 5).

| ***p* value** | Buffer | PL | CL-PL |
| --- | --- | --- | --- |
| Buffer |  | 0.005 | <0.001 |
| PL | 0.005 |  | <0.001 |
| CL-PL | <0.001 | <0.001 |  |


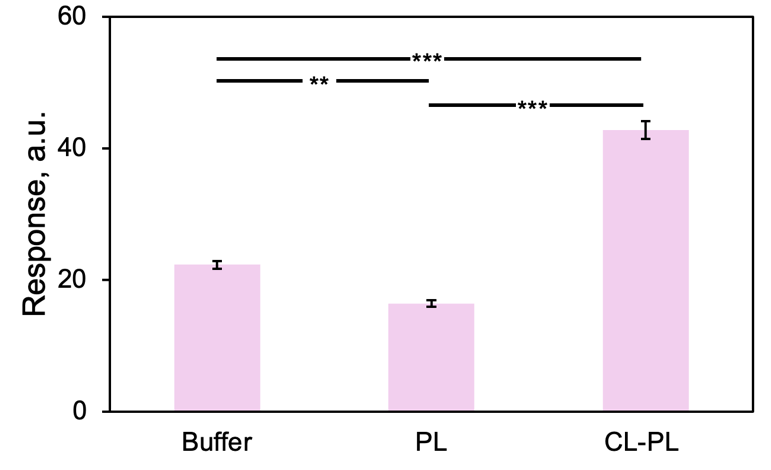


**Fig. S8.** Overall comparison between the means of signal changes during CL-PL, PL and buffer injections (data from Figs. 4 and 5). Error bar stands for the standard error.

- 1. *Comparison between groups and injections (data from Figs. 4 and 5).*

**Table S13.** Descriptive statistics of all groups at all injections (data from Figs. 4 and 5).

| **Injection** | **Groups** | **N** | **Mean** | **Std. Deviation** | **Std. Error** |
| --- | --- | --- | --- | --- | --- |
| Buffer | Background-Sustained | 180 | 16.3151 | 10.41728 | .77646 |
|  | Background-Temporary | 180 | 34.8852 | 8.03194 | .59867 |
|  | Bacteria-Sustained | 260 | 9.9488 | 4.70153 | .29158 |
|  | Bacteria-Temporary | 260 | 34.6054 | 3.89982 | .24186 |
| PL1 | Background-Sustained | 180 | 17.0054 | 3.22057 | 0.24005 |
|  | Background-Temporary | 180 | 9.9337 | 3.62423 | 0.27013 |
|  | Bacteria-Sustained | 260 | 14.2617 | 3.08446 | 0.19129 |
|  | Bacteria-Temporary | 260 | 11.2994 | 3.17229 | 0.19674 |
| PL2 | Background-Sustained | 180 | 3.4767 | 8.80886 | 0.65657 |
|  | Background-Temporary | 180 | 27.9958 | 6.31593 | 0.47076 |
|  | Bacteria-Sustained | 260 | -0.1937 | 4.81637 | 0.29870 |
|  | Bacteria-Temporary | 260 | 35.0958 | 6.17672 | 0.38306 |
| PL3 | Background-Sustained | 180 | -0.2178 | 8.85867 | 0.66029 |
|  | Background-Temporary | 180 | 22.0849 | 11.44027 | 0.85271 |
|  | Bacteria-Sustained | 260 | -4.3145 | 7.99482 | 0.49582 |
|  | Bacteria-Temporary | 260 | 42.2342 | 12.15058 | 0.75355 |
| CL-PL1 | Background-Sustained | 180 | 19.4691 | 3.97167 | 0.29603 |
|  | Background-Temporary | 180 | 12.5492 | 8.13715 | 0.60651 |
|  | Bacteria-Sustained | 260 | 14.1795 | 2.68265 | 0.16637 |
|  | Bacteria-Temporary | 260 | 11.5117 | 3.86061 | 0.23942 |
| CL-PL2 | Background-Sustained | 180 | 2.51 | 5.36 | 0.40 |
|  | Background-Temporary | 180 | 28.90 | 13.04 | 0.97 |
|  | Bacteria-Sustained | 260 | 1.96 | 8.45 | 0.52 |
|  | Bacteria-Temporary | 260 | 36.71 | 10.69 | 0.66 |
| CL-PL3 | Background-Sustained | 180 | 30.234 | 15.007 | 1.119 |
|  | Background-Temporary | 180 | 137.856 | 22.386 | 1.669 |
|  | Bacteria-Sustained | 260 | 41.492 | 19.123 | 1.186 |
|  | Bacteria-Temporary | 260 | 150.776 | 26.314 | 1.632 |

**Table S14.** One-Way ANOVA comparing background and bacteria signals for sustained and temporary responses at each injection (data from Figs. 4 and 5).

| **Injection** | **Background vs Bacteria** | ***p* value** |
| --- | --- | --- |
| Buffer | Sustained | <0.001 |
|  | Temporary | 1.000 |
| PL1 | Sustained | <0.001 |
|  | Temporary | <0.001 |
| PL2 | Sustained | <0.001 |
|  | Temporary | <0.001 |
| PL3 | Sustained | <0.001 |
|  | Temporary | <0.001 |
| CL-PL1 | Sustained | <0.001 |
|  | Temporary | 0.161 |
| CL-PL2 | Sustained | 1.000 |
|  | Temporary | <0.001 |
| CL-PL3 | Sustained | <0.001 |
|  | Temporary | <0.001 |

**Table S15.** One-Way ANOVA results for the sustained signal change from all injections at bacteria area (data from Figs. 4 and 5)

| ***p* value** | buffer | PL 1 | PL 2 | PL 3 | CL-PL 1 | CL-PL 2 | CL-PL 3 |
| --- | --- | --- | --- | --- | --- | --- | --- |
| buffer |  | <0.001 | <0.001 | <0.001 | <0.001 | <0.001 | <0.001 |
| PL1 | <0.001 |  | <0.001 | <0.001 | 1.000 | <0.001 | <0.001 |
| PL2 | <0.001 | <0.001 |  | <0.001 | <0.001 | 0.131 | <0.001 |
| PL3 | <0.001 | <0.001 | <0.001 |  | <0.001 | <0.001 | <0.001 |
| CL-PL1 | <0.001 | 1.000 | <0.001 | <0.001 |  | <0.001 | <0.001 |
| CL-PL2 | <0.001 | <0.001 | 0.131 | <0.001 | <0.001 |  | <0.001 |
| CL-PL3 | <0.001 | <0.001 | <0.001 | <0.001 | <0.001 | <0.001 |  |

**Table S16.** One-Way ANOVA results of the temporary signal change from all injections at bacteria area (data from Figs. 4 and 5)

| ***p* value** | buffer | PL 1 | PL 2 | PL 3 | CL-PL 1 | CL-PL 2 | CL-PL 3 |
| --- | --- | --- | --- | --- | --- | --- | --- |
| buffer |  | <0.001 | 1.000 | <0.001 | <0.001 | 1.000 | <0.001 |
| PL 1 | <0.001 |  | <0.001 | <0.001 | 1.000 | <0.001 | <0.001 |
| PL 2 | 1.000 | <0.001 |  | <0.001 | <0.001 | 1.000 | <0.001 |
| PL 3 | <0.001 | <0.001 | <0.001 |  | <0.001 | <0.001 | <0.001 |
| CL-PL 1 | <0.001 | 1.000 | <0.001 | <0.001 |  | <0.001 | <0.001 |
| CL-PL 2 | 1.000 | <0.001 | 1.000 | <0.001 | <0.001 |  | <0.001 |
| CL-PL 3 | <0.001 | <0.001 | <0.001 | <0.001 | <0.001 | <0.001 |  |


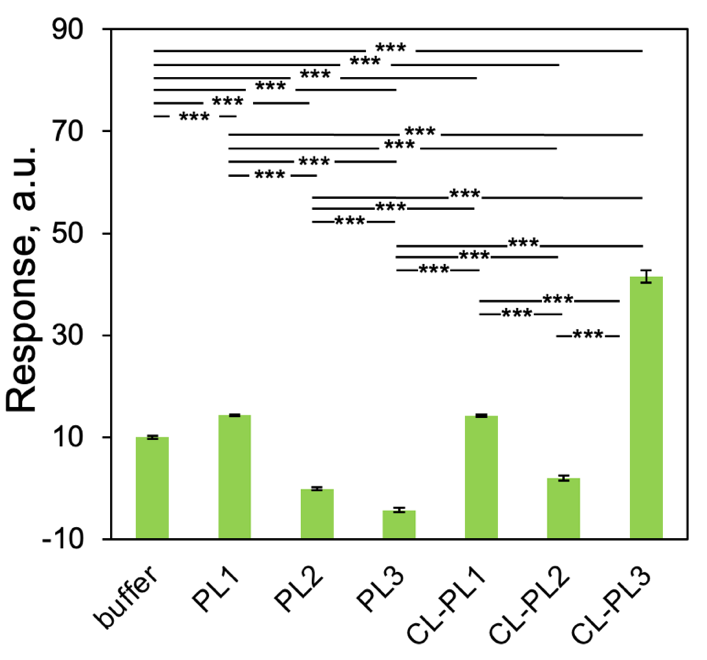

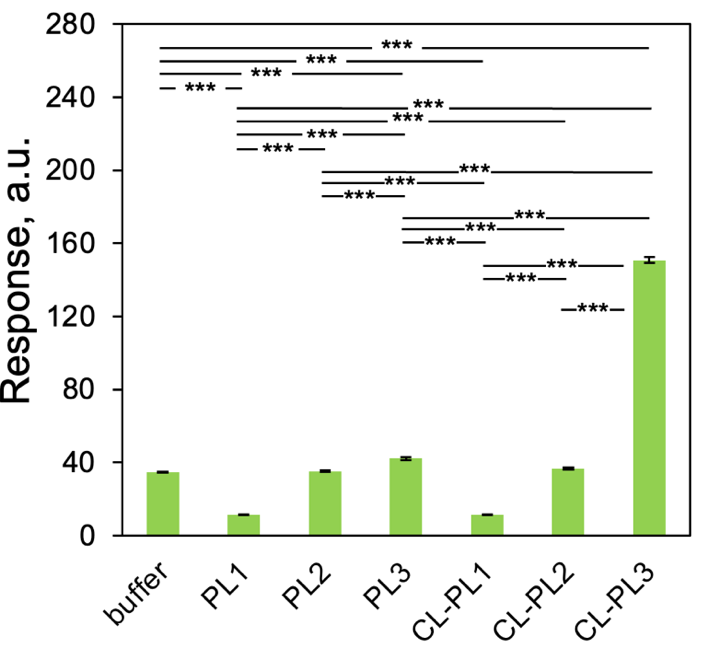


**A**

**B**

**Fig. S9.** Comparison between the means of (A) sustained and (B) signal changes from all injections at bacteria area (data from Figs. 4 and 5). Error bar stands for the standard error.

1. **MATLAB codes for SPRm image processing**

numFrames = 368;

startIndex = 1;

datasetName = "071124 2nd round 8.5 CLPL";

% Initialize images

[imgs, imgsSubFI] = process(numFrames, startIndex);

% Generate imgs + video of imgs - baseline pixel

imgsSubBL = subBL(imgsSubFI, 350, 400, 1, 1); % Ensure imgsSubFI is properly calculated

video = imgsToVid(imgsSubBL, startIndex, numFrames, 1, (datasetName + "subBL_video_br150"), 0, 100);

% Generate imgs + video of imgs - baseline area

imgsSubBL = subBL(imgsSubFI, 600, 450, 5, 5); % Recalculate imgsSubBL

video = imgsToVid(imgsSubBL, startIndex, numFrames, 1, (datasetName + "subBL_area_video_br150"), 0, 100);

% [imgs, imgsSubFI] = process(numFrames, startIndex);

% generate imgs + vid of imgs - baseline pixel

%imgsSubBL = subBL(imgsSubFI, 350, 400, 1, 1);

video = imgsToVid(imgsSubBL, startIndex, numFrames, 1, (datasetName + "subBL_video_br150"), 0, 100);

% generate imgs + vid of imgs - baseline area

imgsSubBL = subBL(imgsSubFI, 600, 450, 5, 5);

video = imgsToVid(imgsSubBL, startIndex, numFrames, 1, (datasetName + "subBL_area_video_br150"), 0, 100);

ip_pixel = zeros(size(imgs,3), 1);

ip_area = zeros(size(imgs,3), 1);

for i=1:size(imgs,3)

ip_pixel(i) = imgsSubFI(50, 50, i);

area = imgsSubFI(1:1+5-1, 1:1+5-1, i);

avg = mean(area(:));

ip_area(i) = avg;

end

%% load images

function [processedImgs, imgsSubFI] = process(numFrames, startFrame)

% get files from current directory

dirName = uigetdir;

filelist = dir([dirName,'\','*.tif']);

% get first image and initialize empty array of images

firstImg = single(imread([dirName,'\', filelist(startFrame).name]));

imgsRaw = zeros(size(firstImg,1),size(firstImg,2),numFrames,'single');

a = waitbar(0, 'Wait for loading images');

%load images

for i = 1:numFrames

waitbar(i/numFrames, a, 'Image Read');

imgsRaw(:,:,i-startFrame+1) = single(imread([dirName,'\', filelist(i).name]));

end

close(a);

imgsSmooth = smoothImgs(imgsRaw);

processedImgs = derivImgs(imgsSmooth);

imgsSubFI = subFI(imgsSmooth);

end

%% subtract baseline

function imgsSubBL = subBL(imgs, bl_i, bl_j, w, h)

imgsSubBL = zeros(size(imgs));

for i=1:size(imgs,3)

area = imgs(bl_i:bl_i+h-1, bl_j:bl_j+w-1,i);

avg = mean(area(:));

imgsSubBL(:,:,i) = imgs(:,:,i)-avg;

end

end

% NOTE: avg = mean(imgs(bl_i:bl_i+h-1, bl_j:bl_j+w-1));

% a = [1, 2, 3, 4; 5, 6, 7, 8; 9, 10, 11, 12]

% i = 1; j = 3; w = 2; h = 3;

% a(i:i+h-1,j:j+w-1)

%% save images as video

function video = imgsToVid(imgs, startIndex, endIndex, step, defaultName, rangeL, rangeH)

videoName = defaultName + ".mp4";

video = VideoWriter(videoName,'MPEG-4'); %create the video object

open (video); %open the file for writing

videoFigure=figure;

for i = startIndex:floor(endIndex/step)

imshow(imgs(:,:,i*step), [rangeL rangeH]);

title(i*step);

colormap("jet"); colorbar;

set(gcf,'color','w');

axis equal;

writeVideo(video,getframe(videoFigure)); % comment for display

end

close(video);

end

%% image smoothing (time & space)

function imgsSmoothSpace = smoothImgs(imgs)

imgsSmoothTime = zeros(size(imgs,1), size(imgs,2), size(imgs,3));

imgsSmoothSpace = zeros(size(imgs,1), size(imgs,2), size(imgs,3));

%smooth on the time domain // remove noise

for i = 1:size(imgs,1)

for j = 1:size(imgs,2)

% smooth fn matlab - types/parameters

imgsSmoothTime(i,j,:) = smooth(imgs(i,j,:),5); %THE FIRST AVERAGE NUMBER

end

end

%smooth on the space domain

for i = 1:size(imgs,3)

imgsSmoothSpace(:,:,i) = filter2(ones(5,5),imgsSmoothTime(:,:,i))/25;

end

end

%% image derivative (+ smoothing)

function imgsSmoothDeriv = derivImgs(imgs)

imgsDeriv = zeros(size(imgs,1), size(imgs,2), size(imgs,3)-1);

imgsSmoothDeriv = zeros(size(imgs,1), size(imgs,2), size(imgs,3)-1);

% derivative

for i = 1:size(imgs,3)-1

imgsDeriv(:,:,i)=imgs(:,:,i+1)-imgs(:,:,i);

end

%smoothing (derivatives)

for i = 1:size(imgs,1)

for j = 1:size(imgs,2)

imgsSmoothDeriv(i,j,:) = smooth(imgsDeriv(i,j,:),15); %THE SECOND AVERAGE NUMBER // final output

end

end

end

%% images subtracted from first image

function imgsSubFI = subFI(imgs)

firstImg = imgs(:,:,1);

imgsSubFI = zeros(size(imgs,1), size(imgs, 2), size(imgs,3));

for i = 1:size(imgs,3)

imgsSubFI(:,:,i) = imgs(:,:,i)-firstImg;

end

end

1. **R code for making violin plots**

library(tidyverse)

library(readxl) # for reading Excel files

# Load the Excel file (select manually)

file_path <- file.choose()

data <- read_excel(file_path)

# Rename columns to match the factor levels used later

colnames(data)[1:4] <- c("total.background", "asso.background", "total.cell", "asso.cell")

# Reshape the data from wide to long format

data_long <- data %>%

pivot_longer(cols = everything(),

names_to = "Category",

values_to = "Value")

# Reorder the Category factor levels

data_long$Category <- factor(data_long$Category,

levels = c("total.background", "asso.background", "total.cell", "asso.cell"))

# Create the violin plot

ggplot(data_long, aes(x = Category, y = Value, fill = Category)) +

geom_violin(trim = FALSE, color = "black") +

scale_fill_manual(values = c("cyan", "deepskyblue3", "beige", "coral2")) +

stat_summary(fun = median, geom = "crossbar",

aes(ymin = ..y.., ymax = ..y..),

color = "black", width = 0.1, size = 0.5) +

labs(title = "071124-8.5clpl",

x = "Category", y = "mean change in response unit") +

theme_minimal() +

theme(

axis.text.x = element_blank(),

plot.title = element_text(hjust = 0.5, size = 20)

) +

scale_y_continuous(limits = c(-150,2500), breaks = seq(-150, 2500, by = 500)) +

annotate("text", x = 1.5, y = -120, label = "Background", size = 5) +

annotate("text", x = 3.5, y = -120, label = "Cell", size = 5)
